# Low Ecotoxicological Impact of Magnesium Oxychloride Cement Composites Doped with 2D Carbon-Based Nanoadditives

**DOI:** 10.1021/acsomega.5c04437

**Published:** 2025-07-31

**Authors:** Simona Lencova, Jana Kofronova, Vaclav Peroutka, Anna-Marie Lauermannova, Adela Jirickova, Michal Lojka, Radek Vurm, Ondrej Jankovsky

**Affiliations:** † Department of Biochemistry and Microbiology, 52735 University of Chemistry and Technology, Prague 166 28, Czech Republic; ‡ Department of Inorganic Chemistry, University of Chemistry and Technology, Prague 166 28, Czech Republic; § Department of Environmental Chemistry, University of Chemistry and Technology, Prague 166 28, Czech Republic

## Abstract

Magnesium oxychloride cement (MOC) is gaining attention as a sustainable alternative to Portland cement. Its mechanical performance and water resistance may be enhanced by reinforcement with two-dimensional nanomaterials, such as graphene (G) and graphene oxide (GO). However, the ecotoxicological impact of these composites, determining their implementation, remains largely unexplored. This study evaluated the effects of G platelets with a surface area of 750 m^2^/g (G750) and GO, both as isolated particles and embedded within MOC, on a range of prokaryotic (*Staphylococcus aureus*, *Escherichia coli*, and *Pseudomonas aeruginosa*) and eukaryotic (*Artemia salina*, *Sinapis alba*, and *Desmodesmus subspicatus*) model organisms. G750 and GO exhibited species-specific antibacterial activity, notably inhibiting *S. aureus* growth and biofilm formation, while *P. aeruginosa* remained largely unaffected. The addition of G750 or GO did not enhance MOC’s antibacterial effect, as MOC alone exhibited strong antimicrobial activity. Both G750 and GO were toxic to *A. salina* at concentrations of ≥0.05 g/L, with GO showing greater toxicity. Phytotoxic effects were observed in *S. alba*, particularly with the GO and MOC-G750 composites. Algal growth was unaffected by MOC-G750 but inhibited by MOC-GO after extended exposure. G750, GO, and MOC samples showed no genotoxic potential *in vitro* and *in vivo*; ROS production occurred without a significant change from the control. Overall, incorporating G750 and GO into MOC improved material properties without substantially increasing ecotoxicity, though species- and material-specific responses underscore the need for thorough environmental impact evaluation.

## Graphical abstract

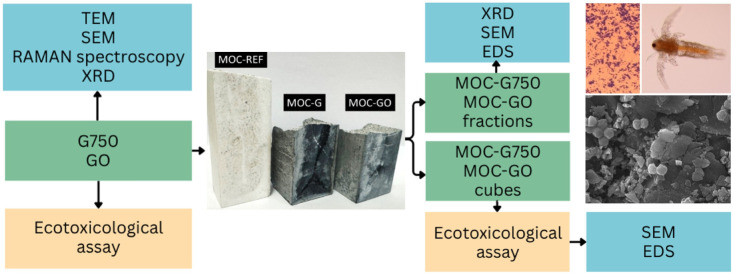

## 1 Introduction

Magnesium oxychloride cement (MOC) has recently been thoroughly researched as an environmentally friendly alternative to commonly used construction binders. The environmental benefits of this material are related to a lower volume of emissions released during its production compared to conventional binders. Furthermore, its ability to sequester atmospheric carbon dioxide while forming chlorocarbonate phases also contributes to its eco-friendly character.^1–3^ Generally, four phases, which form depending on the ratio of raw materials and the conditions of their reaction, are distinguished.^4,5^ Wang et al.^6^ presented a study in which they explored the options for optimization of the raw material ratio and curing temperature to obtain the highest possible values of mechanical parameters while maintaining sufficient water resistance. They showed that the optimal molar ratio between the raw materials, MgO and MgCl_2_, lies in the range of 6–8, the molar ratio between H_2_O and MgCl_2_ should be in the range of 13–15, and the curing temperature should be between 30 and 50 °C to obtain optimal performance of the material.^6^ Generally, the most studied and used phase is phase 5 (Mg_3_(OH)_5_Cl·4H_2_O), which forms at ambient temperature with the MgO/MgCl_2_ molar ratio equal to 5 and the H_2_O/MgCl_2_ molar ratio equal to 13.^7,8^ This phase manifests excellent mechanical parameters even at early stages, good wear resistance, and low thermal conductivity while being susceptible to modification through the application of various functional additives.^9,10^

Modification of MOC is a very wide topic that covers an extensive range of functional modifiers used for various reasons. The motivation to modify MOC lies mainly in the intention of improving or enhancing some of its features, including its mechanical, thermal, or hygric parameters. Among the modifiers, the group of 2D nanomaterials presents an interesting opportunity, showing promising results. It has been previously demonstrated that the excellent structural, mechanical, thermal, and other material properties of 2D nanomaterials^11–14^ translate well into the properties of composites modified by them. Fan et al. described the influence of graphene oxide (GO) on magnesium phosphate cement composites, showing its impact on the mechanical strength and porosity of the resulting composite, which was dependent on the dosage of the nanomaterial.^15^ Furthermore, it was shown that an appropriate amount of GO in magnesium potassium phosphate cement has a positive effect on the hydration reactions occurring during the curing of the composite and therefore, positively influences the microstructure of the resulting composite.^16^ Jiang et al.^17^ explored the effect of graphene (G) on the electrical properties of magnesium sulfate cement, showing an increase in conductivity with the increasing amount of G.^17^ Du et al. studied magnesium potassium phosphate cement composites doped with a hybrid nanomodifier composed of GO and carbon nanotubes, showing their synergistic positive effect on compressive (13.77% increase) and flexural (17.50% increase) strengths when only 0.05 wt % of this hybrid modifier was added.^18^ The use of 2D nanomaterials in MOC was somewhat explored in our previous research. An experiment describing the addition of G and GO into MOC showed their positive influence on mechanical parameters, where G helped to improve the compressive strength. In contrast, the more flexible GO helped to increase the flexural strength. Both of these materials also extensively improved the main flaw of MOC – its water resistance – resulting in softening coefficients comparable to those of conventional construction composites.^19^

Given their advantageous properties, MOC modified with G and GO are considered promising materials for future applications. However, their environmental impact must be thoroughly assessed prior to implementation. Although numerous studies have investigated the effects of G and GO on both prokaryotic and eukaryotic organisms, results remain inconsistent, and no clear consensus has been reached.^20–22^ Considerable focus has been placed on their antimicrobial activity, particularly against Gram-positive and Gram-negative bacteria.^23–25^ Reports on the interaction of *Escherichia coli* and GO suggest, to varying degrees, both insensitivity and bactericidal effects associated with membrane disruption.^24,26^ A generally stronger antibacterial effect has been observed against Gram-positive bacteria,^25^ consistent with the higher resistance of Gram-negative strains due to their complex cell wall structure.^27^ Key variables influencing antibacterial efficacy include particle size, shape, and concentration. For instance, GO’s activity against *E. coli* has been associated with larger nanosheets.^28^ Overall, the physicochemical properties of G materials (e.g., surface area, degree of oxidation, and dispersibility), along with bacterial types and exposure conditions, are critical determinants of their antimicrobial performance.

The same view prevails in the case of eukaryotic organisms. Previous studies have found different effects of G and GO on organisms such as algae, plants, and crustaceans. The effects of GO on algae have been extensively researched, as have the effects of G nanoparticle exposure on algae. Higher concentrations (>0.05 g/L) have been reported to result in severe toxicity. The effects of higher concentrations include the disruption of photosynthesis and chlorophyll content, as well as cellular damage and growth inhibition.^29–31^ However, at concentrations of less than 0.01 g/L, these particles have a lower negative impact and can even improve algal growth in some cases.^32–34^ In plants, GO and G particles generally induce delays in seed germination and inhibit seedling growth with changes of morphology, particularly at elevated concentrations.^35^ For instance, significant inhibition was observed for rice seeds at 0.05 g/L of G,^36^ wheat seeds at 1 g/L of GO,^37^ maize seeds at 0.5 g/L of GO,^38^ or cabbage seeds at 0.5 g/L of G.^39^ In comparison, a lower concentration (0.04 g/L) of GO and G has been shown to enhance the germination of seeds, likely attributable to an enhancement in water transportation.^40,41^ In crustaceans, GO and G can significantly affect behavior in several ways, such as immobilization,^42^ feeding behavior,^43,44^ or physiological changes.^45^ As an example, after exposure to GO, *Daphnia magna* exhibited oxidative stress responses and decreased feeding activity, as observed by Fekete-Kertész et al.,^43^ the feeding activity was only partially restored after a 24-h recovery.

Published studies implicate the need for further research, as G and GO impacts are highly material- and species-specific; thus, each material’s effects must be verified prior to application. Also, while the ecotoxicological effects of G and GO have been studied, their behavior when integrated into building materials, such as MOC, remains largely unexplored. The aim of this study is to evaluate the biological interactions and potential ecotoxicological impacts of graphene (G750), GO, and MOC composites reinforced with these materials. To achieve this, we investigate their effects on a diverse set of model organisms selected for their environmental relevance and importance in ecotoxicology and human health, including *Staphylococcus aureus*, *E. coli*, *Pseudomonas aeruginosa*, *Artemia salina*, *Sinapis alba*, and *Desmodesmus subspicatus*. This multifaceted approach provides a comprehensive assessment of the ecological risks associated with the use of graphene-based additives in cementitious materials.

## 2 Materials and Methods

### 2.1 Raw Materials

For the preparation of MOC-based composites with carbon nanomaterials, MgO powder with 98% purity obtained from Penta, s.r.o. (Prague, Czech Republic), MgCl_2_·6H_2_O of analytical grade sourced from Lach-Ner, s.r.o. (Neratovice, Czech Republic), water, and three size fractions of silica sand – PG1 (0.0–0.5 mm), PG2 (0.5–1.0 mm), and PG3 (1.0–2.0 mm) – supplied by Filtran psky Ltd. (Chlum u Doks, Czech Republic) were used. As the nanomodifiers, G platelets with a surface area of 750 m^2^/g (G750) purchased from Sigma-Aldrich (USA) and graphene oxide (GO) of industrial grade supplied by ACS Material LLC (Pasadena, USA) were used. The purchased G and GO were analyzed using TEM, SEM, Raman spectroscopy, and XRD; details on instrumentation and settings are available in previous publications.^46^

### 2.2 Sample Preparation and Characterization

A reference material, MOC-REF, was prepared without G750 and GO for comparison. The composite samples, MOC-G750 and MOC-GO, contained their respective nanodopants at a concentration of 1.0 wt % relative to the cement paste mass (combined mass of MgO, MgCl_2_·6H_2_O, and H_2_O). Table 1 presents the composition of the prepared MOC composites. To prepare the MOC samples, MgCl_2_·6H_2_O was dissolved and combined with MgO powder and silica sand by using a planetary mixer. For the MOC-based composites, the respective nanodopant was first homogenized using an Ultra Turrax (IKA, Germany) in the MgCl_2_·6H_2_O solution before mixing with MgO and silica sand. The wet mixtures were then poured into prismatic molds with dimensions of 40 mm × 40 mm × 160 mm. After 1 day, the samples were demolded and left to cure in air for 27 additional days. One part of each of the prepared composite samples was crushed and analyzed using XRD and SEM. Another part was cut into small cubes (1 cm × 1 cm × 1 cm), crushed into powder, and used for ecotoxicity tests. Furthermore, samples that underwent exposure to the prokaryotes were further analyzed using SEM and EDS. A more detailed preparation procedure and details on instrumentation and settings can be found in previously published works.^19,46^

**Table 1:** Mixture Composition of MOC-REF, MOC-G750, and MOC-GO (in g)

Sample	MgO	MgCl_2_·6H_2_O	H_2_O	G	GO	Silica sand
**MOC-REF**	100.0	100.9	62.6	-	-	3 × 100.0
MOC-G750	100.0	100.9	62.6	2.6	-	3 × 100.0
**MOC-GO**	100.0	100.9	62.6	-	2.6	3 × 100.0

### 2.3 Bacterial Strains, Eukaryotic Organisms, and Culture Conditions

As model bacterial strains, the Gram-positive bacterium *Staphylococcus aureus* ATCC 25923 (eq CCM 3953) and the Gram-negative bacteria *Escherichia coli* ATCC 25922 (eq CCM 3954) and *Pseudomonas aeruginosa* ATCC 27853 (eq CCM 3955), obtained from the Czech Collection of Microorganisms (CCM, Czech Republic), were used; these strains serve as international reference strains for testing antibacterial activity. Pure bacterial suspensions in tryptone soy broth (TSB, Oxoid Ltd., Great Britain) were stored in a mixture with glycerol (25%) in a freezer (−80 °C). For recultivation, suspensions were inoculated into sterile TSB (10 mL) and incubated at 37 °C for 24 h. After the incubation, suspensions were centrifuged (5 min, 5000 *g*), and the obtained pellet was resuspended in sterile TSB. The optical density of the fresh suspension was adjusted to 0.5 McFarland and used for the ecotoxicological assay.

As model eukaryotic organisms relevant for ecotoxicological studies, aquatic crustaceans *Artemia salina* (EasyFish, Czech Republic), mustard *Sinapis alba* (Biom, Czech Republic), and algae *Desmodesmus subspicatus* (Culture Collection of Autotrophic Organisms, Czech Republic) were used.

### 2.4 Ecotoxicological Assay – GO and G750

#### 2.4.1 Impact on Bacterial Growth and Biofilm Formation

First, the effect of G750 and GO at three different concentrations (1, 0.1, and 0.01 g/L) on **bacterial growth** was tested. The mixtures containing bacteria, TSB, and G750/GO were continuously orbitally mixed, and the absorbance was continuously measured spectrophotometrically at λ = 600 nm for 24 h at 37 °C on a BioTek Synergy H1 Multimode Reader (Agilent, USA). After deducting blank values, growth curves were compiled, and the maximum specific growth rate was determined by linear regression of five consecutive values in the exponential phase of the curve.

Next, the effect of G750 and GO on bacterial **biofilm formation** was investigated. The biofilm was cultivated in a 96-well microtiter plate (Gama Group, Czech Republic) under the following conditions. Each well contained 160 μL of sterile TSB, 20 μL of a single-species bacterial suspension (0.5 McF), and 20 μL of G750/GO solution in TSB to achieve a final concentration of 1, 0.1, or 0.01 g/L. Control samples for microbial growth contained 180 μL of TSB and 20 μL of bacterial suspension (0.5 McF). Control samples with the G750/GO medium contained 180 μL of TSB and 20 μL of the G750/GO-containing TSB solution. The plates were incubated at 37 °C for 24 h. After cultivation, the biofilm was washed five times with a saline solution (0.9% NaCl in distilled water), dried for 45 min at room temperature, and then stained with 0.1% crystal violet solution (CV, Sigma-Aldrich, USA) for 45 min. The samples were then washed three times with saline solution and analyzed in two ways: (i) microscopically at 60× magnification or (ii) by incubation with 200 μL of 96% ethanol (Penta, Czech Republic) for 15 min at room temperature, followed by transferring 100 μL to a sterile 96-well microtiter plate and spectrophotometric measurement of absorbance at 595 nm (Tecan, Switzerland).

#### 2.4.2 Determination of Toxicity on Eukaryotic Organisms

For all tests performed with eukaryotes, the test samples were prepared in the same way. The powder of the material was dispersed in a control medium specific to each organism and then sonicated in an ultrasonic bath for 30 min. After this time, solutions of different concentrations were prepared and used immediately for tests with different organisms. The concentrations tested ranged from 0.01 to 1 g/L for *A. salina* and *S. alba*, while for *D. subspicatus* only concentrations from 0.01 to 0.1 g/L were used due to possible shielding effects on the results.

The **mortality test for** *A. salina* was performed with respect to standard laboratory procedures. The *Artemia* nauplii used for the test were secured by incubation in salt water at 26 °C in a specialized hatching device (JBL, Germany). After 24 h, the nauplii were transferred to a control medium, which consisted of a demineralized water solution containing 30 g/L sodium chloride. Potassium dichromate was used as the standard. The nauplii were then placed in Petri dishes (10 pcs/dish) and covered with a 10 mL volume of material, control, or standard solution. The dishes were sealed and incubated for 24 h at 20 ± 2 °C in the dark. Tests were performed in triplicate. At the end of the incubation period, mortality was determined, followed by microscopic examination of potential bioaccumulation in the crustacean body using a Primo Star microscope (Zeiss, Germany) at 40× magnification.

The **growth test for mustard** *S. alba* **seeds** was performed with respect to standard laboratory procedures. The seeds were obtained from a local supplier. The control medium and the solution for dispersed particles were prepared by adding aliquots of solutions containing different salts to demineralized water. The standard used was potassium dichromate. The test procedure was as follows: 15 seeds were placed evenly on Petri dishes (120 mm diameter) with filter papers with preprepared holes, soaked with 5 mL of test solutions. The dishes were sealed and placed in an incubator, where they were maintained for 3 days in the dark at 20 ± 2 °C. Three replicates were prepared for each test solution. After the incubation period, the number of germinated seeds was determined, and root length was measured using ImageJ software (National Institutes of Health, USA). The results obtained were then expressed as the germination index (GI), indicating phytotoxicity according to the equation:

$$GI=\frac{N_{S}\times L_{S}}{N_{C}\times L_{C}}\times 100\left[\%\right]$$

where *N*_S_ is the number of germinated seeds in the sample; *N*_C_ is the number of germinated seeds in the control; *L*_S_ is the average root length in the sample; *L*_C_ is the average root length in the control. A value of less than 50% is indicative of high phytotoxicity, values between 50 and 80% suggest slight phytotoxicity, and values between 80 and 100% indicate an absence of phytotoxicity. When the value of GI is more than 100%, it is considered that the substance tested is a phytonutrient or a phytostimulant.^47^

The **algal growth inhibition test on** *D. subspicatus* was performed according to EN ISO 8692. Algae preculture was performed 3 days prior to the start of the bioassay in a flask filled with Bold’s Basal Medium (BBM), sealed with a plug, and placed in an orbital shaker (ELMI DOS-20L, ELMI USA) in an incubator at 23 ± 2 °C under constant illumination (7000 lx). Samples prepared by dispersion in BBM medium, which also served as a control, were pipetted into 15 mL Erlenmeyer flasks and inoculated with algae at an initial concentration of 2 × 10^4^ cells/mL. The flasks were then placed in an incubator and left for 3 days under the same temperature and light conditions as the preculture. Tested samples were prepared in triplicate. After the culture period, the algal concentration was determined using a Primo Star light microscope (Zeiss, Germany) and a Bürker counting chamber at 400× magnification. The growth inhibition rate was calculated from the algal concentrations found. To determine the effect of the materials on algal growth over a longer period, the algae were cultured for 8 days. During this time, their concentrations were continuously monitored. The results were then plotted as growth curves.

### 2.5 Ecotoxicological Assay – MOC Samples

In the ecotoxicological assay, we focused on the overall determination of the influence of MOC-G750 and MOC-GO on selected organisms. First, their impact on bacterial growth and biofilm formation was examined. Regarding **bacterial growth**, pieces of MOC samples (1 × 1 × 1 cm) were incubated in 3 mL of single-species bacterial suspensions (0.5 McF) of *E. coli*, *S. aureus*, and *P. aeruginosa*, respectively, at 37 °C for 48 h. Control samples for bacterial growth contained only 3 mL of bacterial suspension, while sterility controls for MOC included 3 mL of sterile TSB and an MOC piece. Before and after cultivation, the absorbance of the suspension at 620 nm was measured by using a spectrophotometer (Tecan, Switzerland). Additionally, the pH of the suspensions was measured before and after cultivation using a calibrated pH meter (Radiometer Analytical, France).

In the next step, the influence of MOC samples on bacterial **biofilm formation** was examined. MOC pieces (1 × 1 × 1 cm) were incubated under the same conditions as in the growth study: in 3 mL of *E. coli*, *S. aureus*, or *P. aeruginosa* suspensions (0.5 McF) at 37 °C for 48 h, and controls were prepared similarly to the above-mentioned experiment. After cultivation, MOC samples were washed with a saline solution and sonicated in 3 mL of sterile saline solution for 3 min at room temperature using a sonication bath (Polsonic, Poland). The saline solution containing released biofilm-forming cells was then serially diluted in sterile saline solution up to the eighth dilution. Droplets (20 μL) from each dilution were plated in triplicate on Tryptone Soy Agar (TSA, Merck, Germany) and incubated for 24 h at 37 °C. Then, colony-forming units (CFUs) were counted, normalized to 1 cm^2^, and compared with the control. Further, SEM analysis was performed to examine bacteria that adhered to the MOC surface. Energy-dispersive spectroscopy (EDS) was used to confirm the presence of organic matter, following previously published methodologies.^48–50^

Finally, the MOC toxicity on **eukaryotes** was investigated. *A. salina* mortality, *S. alba* growth, and *D. subspicatus* growth in the presence of MOC samples were determined as described in [Sec sec2.4.2].

### 2.6 Genotoxic Potential

Genotoxic potential was assessed *in vitro*. Solutions of G750, GO, and MOC samples at a concentration of 1 g/L, diluted in nuclease-free water (Promega, USA), were mixed with commercially available lambda dsDNA (Promega, USA) at a concentration of 100 ng/μL. After incubation (2 h, 20 °C), horizontal electrophoresis (1% agarose gel, Midori Green (Promega, USA) addition) was performed (90 min, 90 V). Sample staining was performed with Promega 5X Green GoTaq Flexi Reaction Buffer (Promega, USA), and products were visualized under UV light (Quantum Vilber Lourmat, France). For *in vivo* genotoxicity analysis, solutions of G750, GO, and MOC samples were mixed with a fresh suspension of *E. coli* ATCC 25922 (0.5 McF) so that the final concentration of the tested materials was 1 g/L. After incubation (24 h, 37 °C), *E. coli* DNA was isolated using the Invitrogen PureLink Genomic DNA Mini Kit (Thermo Fisher Scientific, USA). The concentration and purity of the DNA were verified spectrophotometrically using a Microvolume UV–vis Spectrophotometer NanoDrop One instrument (Thermo Fisher Scientific, USA). Horizontal electrophoresis and product visualization were performed as described in the *in vitro* analysis.

### 2.7 Reactive Oxygen Species Production

Hydrogen peroxide (H_2_O_2_), a key marker of oxidative stress, was quantified using the ROS-Glo H_2_O_2_ Assay (Promega, USA) according to the manufacturer’s protocol. Luminescence was measured using a BioTek Synergy H1 Multimode Reader (Agilent, USA), and the percentage increase in reactive oxygen species (ROS) production was calculated relative to that in the control group (bacteria without G/MOC exposure). The tests were conducted on model bacterial strains *E. coli*, *S. aureus*, and *P. aeruginosa*.

### 2.8 Data Analysis

To ensure the robustness of the data, all above-described biological experiments were conducted in at least biological and technical triplicates. Data were analyzed as described in our previous study^50^ using the R programming language. Dean-Dixon’s Q-test was used for outliers’ identification and elimination. The Shapiro-Wilk test was applied to assess data distribution normality (data with *p* > 0.05 were considered normally distributed). The data were analyzed by one-way analysis of variance (ANOVA) with significance levels set at α = 0.05, α = 0.01, and α = 0.001. If significant differences were found, Tukey’s Honest Significant Differences (Tukey HSD) and Bonferroni correction were applied for multiple pairwise comparisons of group means. Statistical analysis was conducted to evaluate the initial hypothesis that both tested forms of G, as well as the prepared MOC samples enriched by them, influence bacterial and eukaryotic growth and viability.

## 3 Results and Discussion

To robustly evaluate the properties of G750, GO, and prepared MOC samples and their interactions with model organisms, the workflow described in Figure 1 was followed.

**Figure 1:**
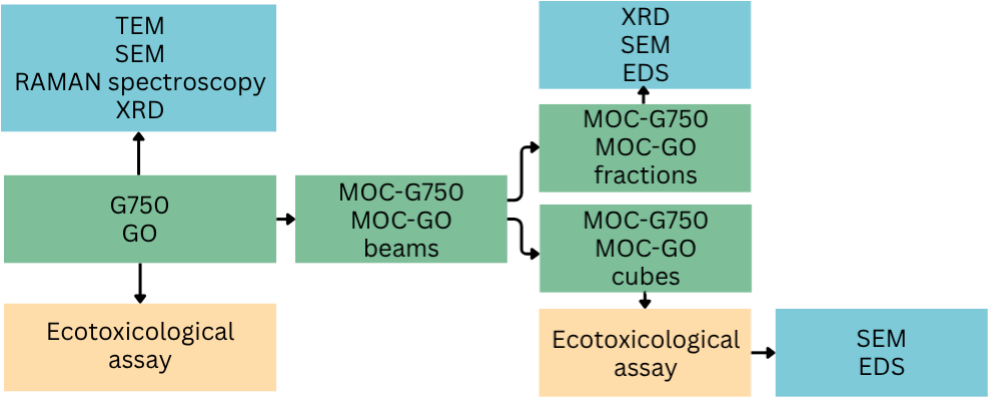
Schematic workflow of the study.

### 3.1 Material Preparation and Characterization

#### 3.1.1 G750 and GO Characterization

The nanomaterials used for the modification of the designed MOC-based composites, G750 and GO, were studied using TEM, SEM, Raman spectroscopy, and XRD. The obtained micrographs from TEM and SEM measurements are shown in Figure 2A,B. The micrographs demonstrate the layered structure, with the sheet size ranging between 2 and 10 μm and a sheet thickness of a few nanometers for both types of nanomodifiers. In Figure 2C, G750 exhibits a characteristic sharp peak at 2θ ≈ 26.5°, corresponding to the (002) plane of graphitic carbon, indicating a high degree of crystallinity and graphitic stacking. In contrast, the GO sample shows a broad peak centered at 2θ ≈ 11.0°, attributed to the (001) reflection of oxidized graphene layers. The significant shift and broadening of the peak in GO confirm the introduction of oxygen-containing functional groups and the increased interlayer spacing due to oxidation, indicating a disrupted graphitic structure. In Figure 2D, both spectra exhibit characteristic D band (∼1350 cm^–1^) and the G band (∼1580 cm^–1^). The G750 sample shows a relatively low D/G ratio (D/G = 0.58), indicating fewer defects and a higher degree of graphitic order. In contrast, the GO spectrum displays a more intense D band and a higher D/G ratio (D/G = 0.96), reflecting the increased presence of structural defects and disordered carbon domains due to oxidation and functionalization. The 2D band (∼2700 cm^–1^) is also more pronounced in G750, suggesting better-preserved stacking of graphene layers, whereas it is diminished and broadened in GO.

**Figure 2:**
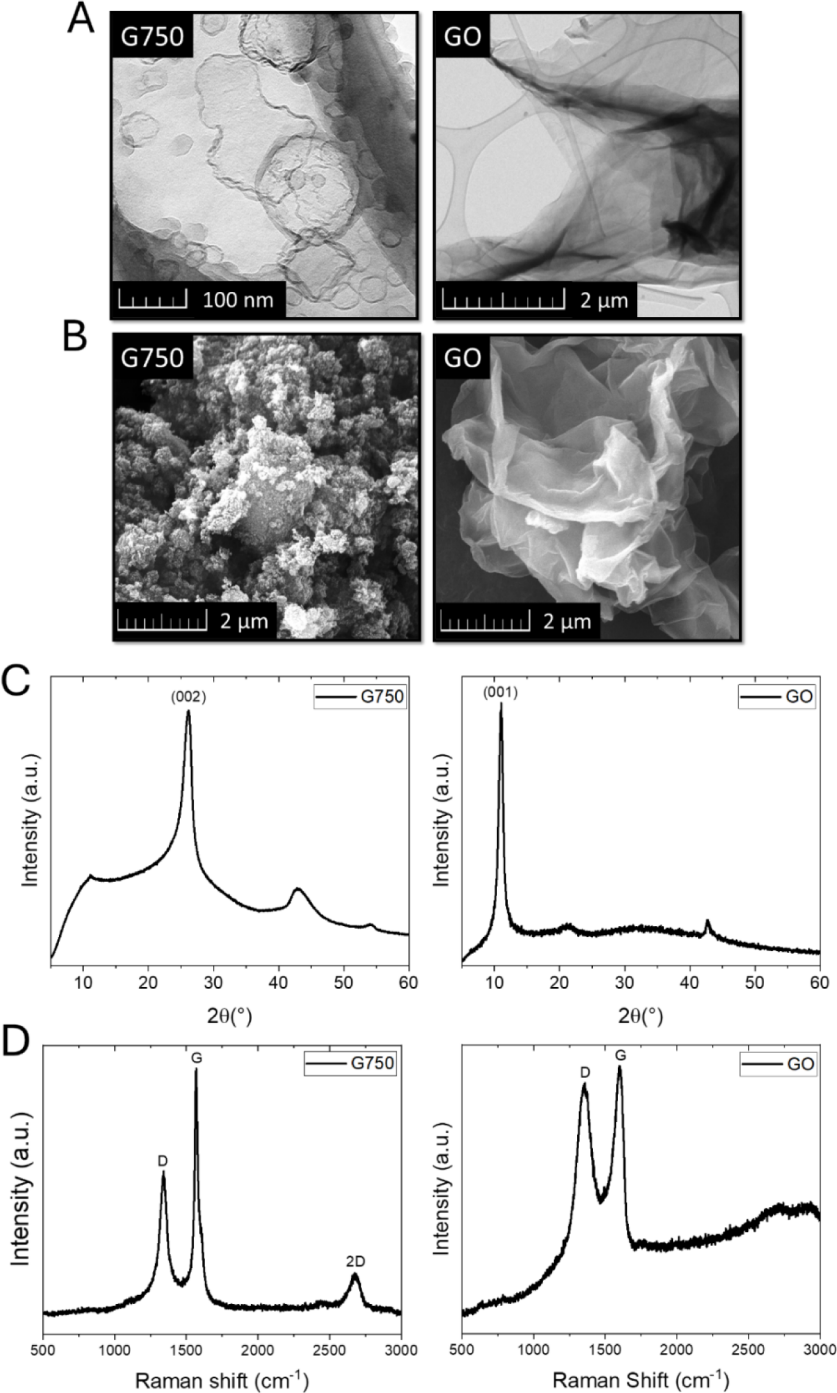
TEM (A), SEM (B), XRD (C), and Raman spectroscopy (D) micrographs of G750 and GO.

#### 3.1.2 MOC Preparation and Characterization

The characterized nanomaterials, G750 and GO, were implemented as nanomodifiers in MOC-based composites filled with silica sand as a filler. Fragments of the prepared samples are shown in Figure S1. The phase composition of the prepared MOC-based composites was studied by using XRD. The obtained diffraction patterns are shown in Figure 3. All samples contained the same crystalline phases – MOC phase 5 (Mg_3_(OH)_5_Cl·4H_2_O, ICDD 04–014–8836) and quartz (SiO_2_, ICDD 01–085–0865). The presence of these phases was also confirmed by microstructural analyses described in the following paragraphs. The presence of the nanomodifiers was not distinguished in the diffraction patterns, mainly due to the low content of these additives.

**Figure 3:**
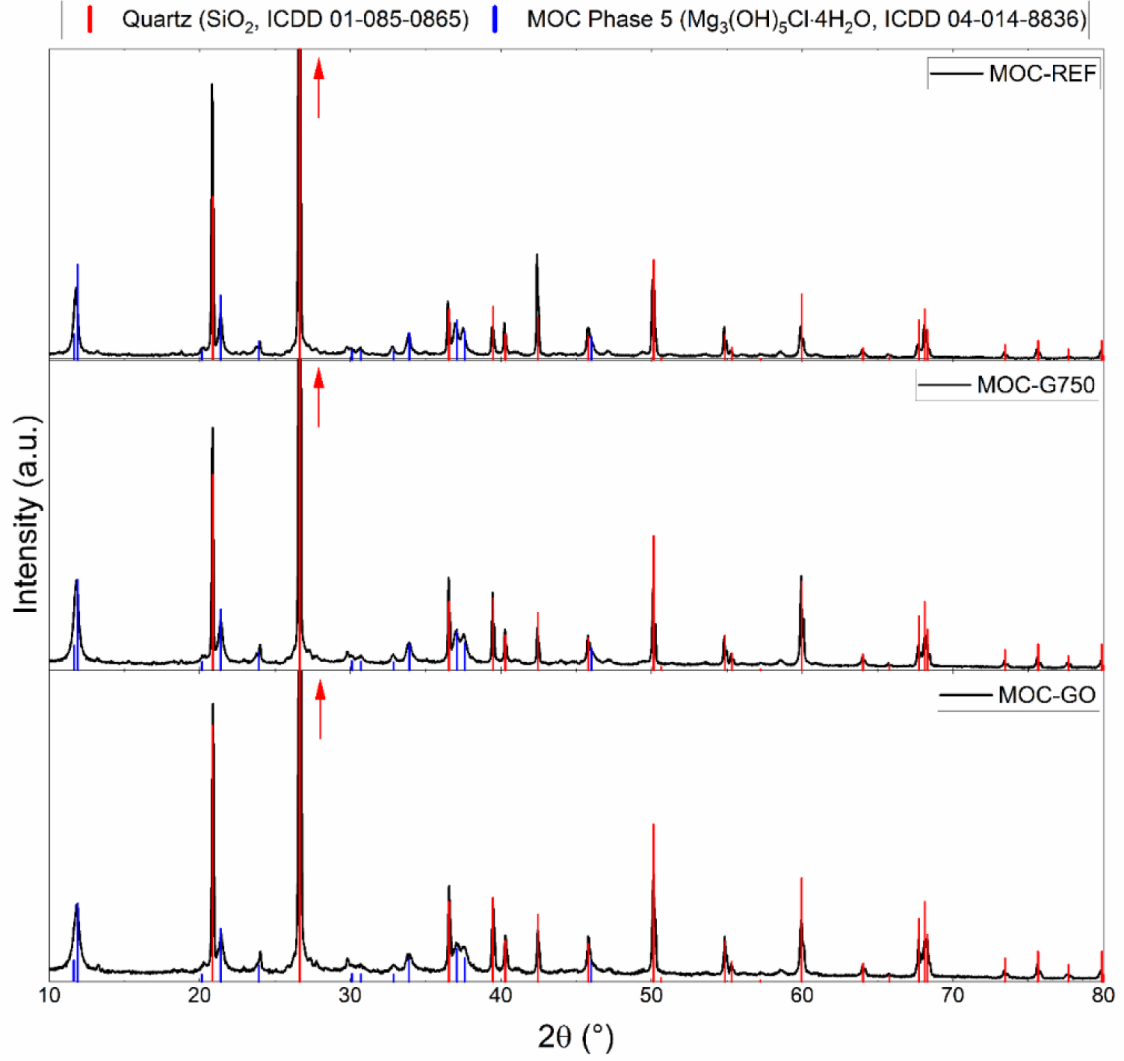
Diffraction patterns of the prepared MOC-based composite samples.

The microstructure and morphology of the fracture surface of the prepared composites were studied by using SEM (Figure 4). All types of prepared samples manifest a dense microstructure consisting of interlocking MOC phase 5 crystals, which is typical for MOC-based composites. Furthermore, grains of the filler, silica sand, can be seen in the micrographs. Due to their size, the particles of G750 are not quite distinguishable in the micrographs; however, at the highest magnification, the GO sheets in the sample MOC-GO are visible very well. At smaller magnifications, some air bubbles formed during the preparation of the samples are visible.

**Figure 4:**
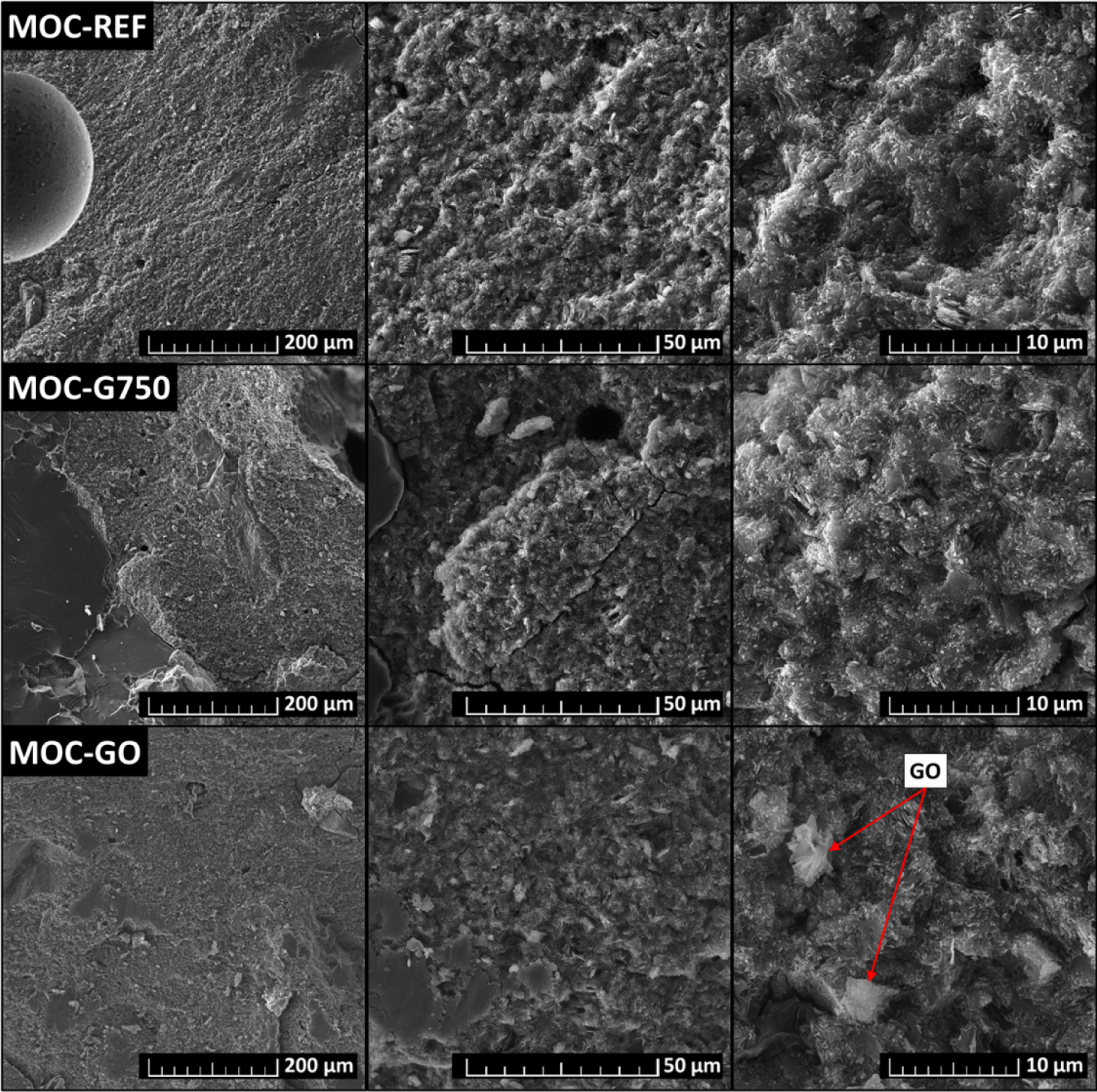
Microstructure of prepared MOC-based samples obtained from SEM.

### 3.2 Impact of G750 and GO on Prokaryotes and Eukaryotes

To assess the impact of G750 and GO on selected organisms, concentrations of 1 g/L, 0.1 g/L, and 0.01 g/L were chosen to represent varying environmental exposure scenarios and ensure comparability with published studies, where these doses are commonly used.^51^ The highest concentration (1 g/L) simulates a worst-case scenario and is frequently applied in dispersion stability tests, as nanomaterial agglomeration and dissolution kinetics depend on concentration. It may also reflect potential bioaccumulation or localized contamination events. Conversely, the lowest concentration (0.01 g/L) approximates environmental levels following industrial runoff or accidental release and is suitable for acute toxicity assessments.

First, the effects of G750 and GO on bacterial growth were assessed via a spectrophotometric analysis of growth curves and maximum growth rates (Figure 5). For these analyses, two concentrations of G750 and GO were selected (0.1 g/L, 0.01 g/L) due to the high absorbance of the higher concentration (1 g/L), disabling accurate spectrophotometric quantification. Model bacterial species were selected to match those commonly used in related studies, facilitating comparison and capturing key differences in bacterial traits—namely, cell wall structure (Gram-positive vs Gram-negative), morphology (cocci: *S. aureus*; rods: *E. coli*, *P. aeruginosa*), and size (*S. aureus*: ∼0.5–1.0 μm diameter; *E. coli*: ∼1.0–2.0 μm long, 0.25–1.0 μm diameter; *P. aeruginosa*: ∼1.5–3.0 μm long, 0.5–0.8 μm diameter). The methodology was selected as the standard approach. While it can be complemented by viability and metabolic assays (e.g., MTT, resazurin), these methods require further optimization for applications involving G materials, as their accuracy is highly dependent on the specific experimental conditions and interactions with these nanomaterials.^52^

**Figure 5:**
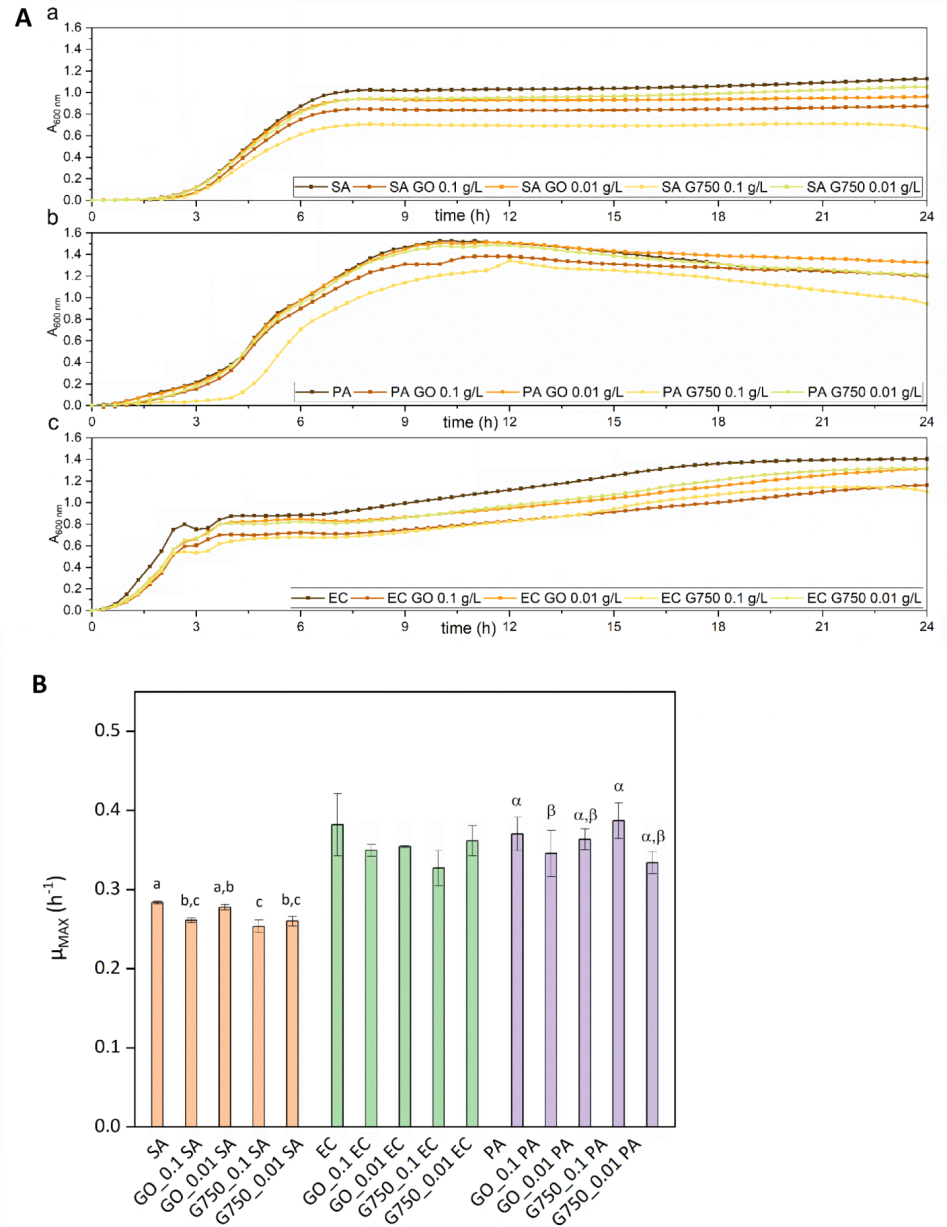
Influence of G750 and GO on bacterial growth: A) growth curves of (a) *S. aureus* (SA), (b) *E. coli* (EC), (c) *P. aeruginosa* (PA) and B) bacterial maximum growth rate in the presence of different concentrations (0.1 g/L, 0.01 g/L) of GO and G750, respectively. Means that do not share a letter within the same column are statistically significant at *p* < 0.05; for EC, no significance at α = 0.05 was found.

Results revealed species-specific responses: *S. aureus* exhibited growth inhibition, particularly at 0.1 g/L for both tested materials, and G750 was the more impactful material than GO regarding its influence on maximum growth rate, whereas Gram-negative bacteria showed minimal changes. *Pseudomonas aeruginosa* was the least affected, aligning with its known resistance to external stressors.^53^ The species-specific responses correspond to the known higher resilience of Gram-negative bacteria to external stressors, attributed to their distinct cell wall structure compared to Gram-positive ones.^25–27^ Further, both G materials inhibited biofilm formation across all concentrations, which may be partially explained by the settling of G particles, preventing bacterial adhesion to the bottom, with species-specific responses observed (Figure 6). The physicochemical interactions between bacterial cell surfaces and G materials likely contribute to these differences; for example, variations in cell surface charge and hydrophobicity among species can affect attachment strength and biofilm architecture. *S. aureus* was the most sensitive one, mirroring growth inhibition results, while *P. aeruginosa* showed the lowest susceptibility, with no significant inhibition by G750 at the concentrations of 0.1 or 0.01 g/L. *S. aureus* may be at a disadvantage compared to *E. coli* and *P. aeruginosa* due to its tendency to quickly form grape-like clusters, which limits its access to free space on the material. *P. aeruginosa* also tends to form microcolonies, particularly when any seeding space is available; however, cell aggregation kinetics are usually slower compared to *S. aureus*. This difference in aggregation rates can be attributed to various factors, including the distinct mechanisms and regulatory pathways governing biofilm formation in these species.^54^ Individual cells, in contrast, can avoid G particles more easily and colonize the available areas.

**Figure 6:**
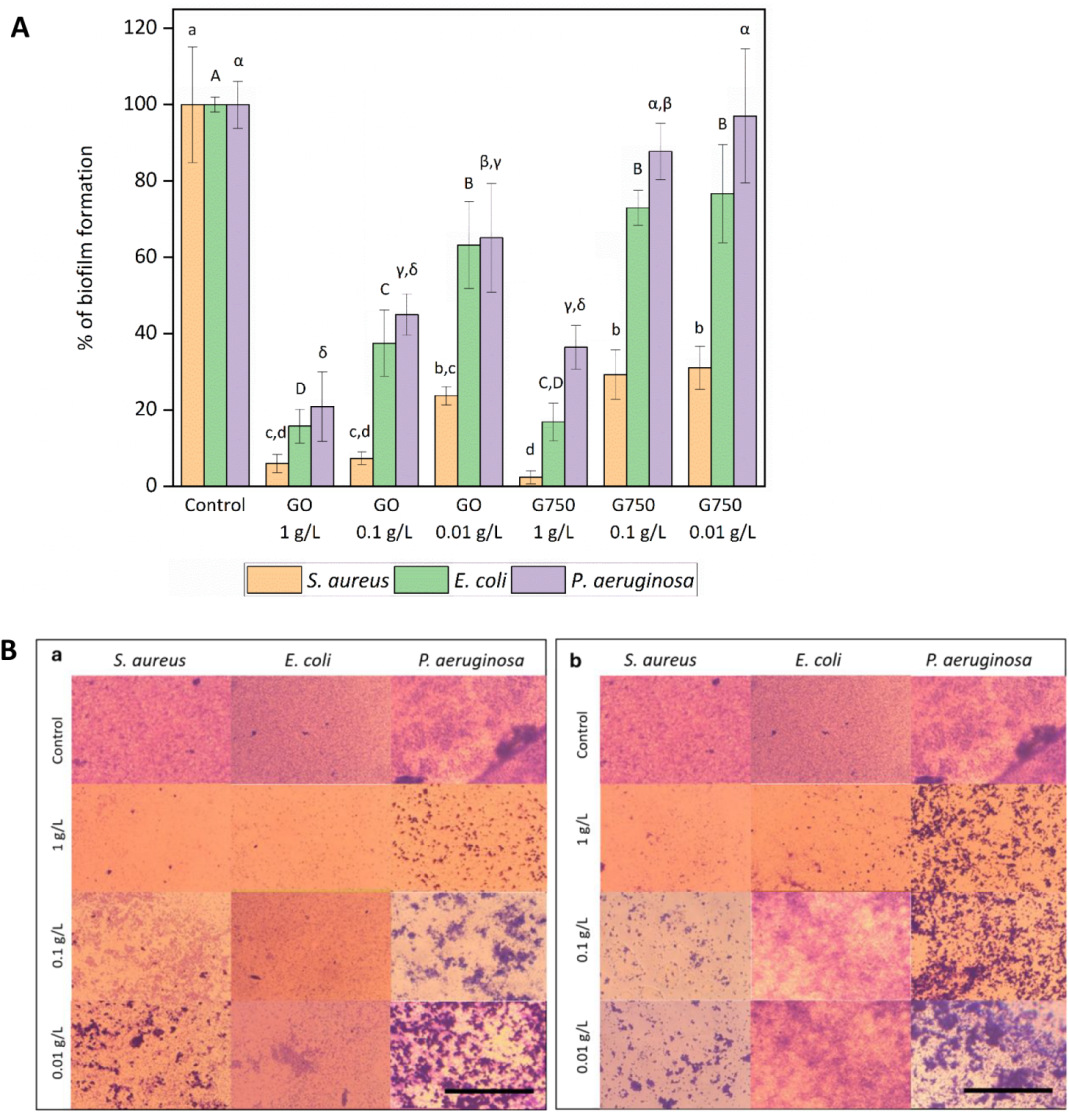
Influence of GO and G750 (1, 0.1, and 0.01 g/L) on bacterial biofilm formation determined via CV staining: A) quantitative analysis (means that do not share a letter within the same column are statistically significant at *p* < 0.05), B) qualitative analysis, scale bar = 200 μm; biofilm appears violet—more purple biomass reflects lower antibiofilm activity of the tested materials.

This observation supports the general findings from other studies, confirming the G750 and GO antimicrobial potential. Several studies have reported a strong antimicrobial effect against *S. aureus* and *E. coli*, which are usually tested as model bacteria in the literature,^23–25^ and highlighted the potential of G-based materials for effective antibiofilm coatings.^24^ The antibiofilm activity of the tested materials is highly beneficial in building material preparation, as microbial degradation causes significant damage.^55^ However, the findings across studies remain inconsistent, making it difficult to clearly define the presence or strength of the antibacterial effects of these materials. Although it has been reported that GO is more effective against *S. aureus* compared to *E. coli*,^56^ some studies have concluded the quite opposite species-specific trend. For instance, it was demonstrated that GO-coated surfaces inhibited biofilm formation more effectively in *E. coli* than in *S. aureus*.^57^ Further, Akhavan and Ghaderi^23^ reported no antibacterial effect—a tested *E. coli* strain showed resistance to GO. While our results support the majority of studies indicating antimicrobial effects, the outcome depends on multiple factors—physicochemical properties of G materials, tested concentrations (generally comparable across studies), and species- or strain-specific bacterial responses.

As prokaryotic and eukaryotic organisms fundamentally differ in their physiology, different responses to external factors and stressors can be presumed. To complement the analysis of the influence of G750 and GO samples on bacteria, we further investigated their impact on *A. salina*, *S. alba* and *D. subspicatus*. These organisms represent environmentally highly relevant species and well-established, widely accepted models for ecotoxicological assays.

Saltwater crustaceans *A. salina* were exposed to concentrations of GO and G750 ranging from 0.01 to 0.1 g/L. For both tested nanomaterials, the concentration from 0.05 g/L caused 100% mortality (Figure 7). A lower concentration of 0.025 g/L showed a different result, where GO caused mortality significantly higher than that of G750. The difference at the lowest tested concentration was not significant. Our study contrasts with that of Mesarič et al.,^58^ who found no effect on *A. salina* larvae after being exposed to GO at a concentration of 0.6 g/L, but 90% mortality was observed at a concentration of 0.7 g/L, along with oxidative stress biomarkers and changes in swimming behavior. In another study, a significant increase in mortality of about 80% was observed at a GO concentration of 0.6 g/L, but at 0.05 g/L, mortality was already below 10%. This study also reported body damage and accumulation of GO in the gut.^59^ Another study obtained the same results for mortality in high concentrations, however similar results to ours in terms of negative effects due to oxidative stress were observed as early as 0.001 g/L.^60^ The potential explanations for the observed variations in outcomes may be attributed to factors such as different experimental conditions, exposure duration, aggregation state, and also characteristics of GO such as the sheet size, thickness, and oxidation degree. For G particles, no harmful effect on *A. salina* at a concentration up to 0.01 g/L was found in another recent study.^61^ This finding is in accordance with the results of our study, and moreover, the results are consistent with each other, where higher toxicity was observed for GO rather than G. The majority of studies focus on GO; nevertheless, the effects on *A. salina* may be comparable, albeit to a different degree.

**Figure 7:**
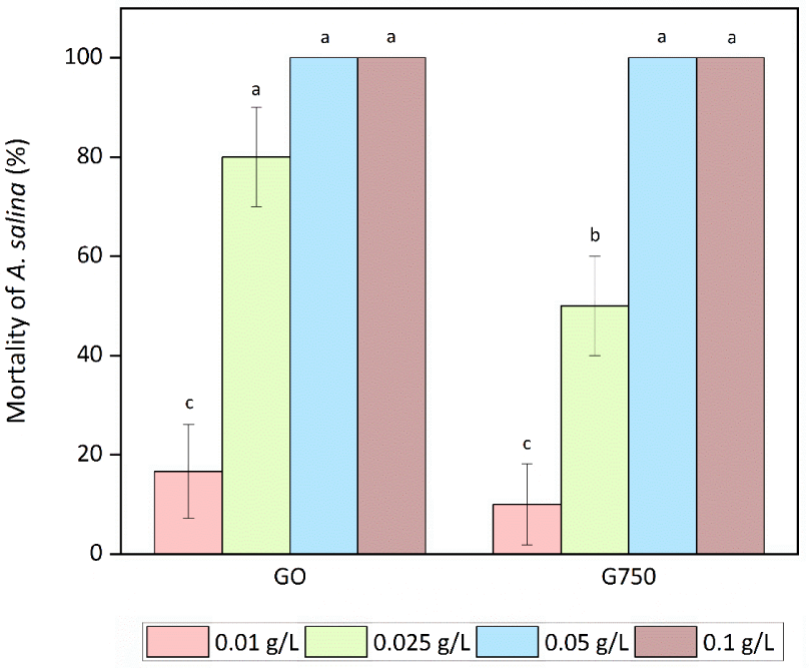
*A. salina* mortality after exposure to GO and G750 for 24 h. Means that do not share a letter are statistically significant at *p* < 0.05 (ANOVA and Tukey’s test with Bonferroni correction).

The results of the germination index (GI) for *S. alba* seeds are shown in Table 2. GI values higher than 80% indicate no phytotoxicity, while lower values are attributed to a phytotoxic effect. In the absence of toxicity observed at a concentration of 0.01 g/L for GO, the presence of inhibition at a concentration of 0.1 g/L is indicative of a potential phytotoxic effect. Conversely, no significant phytotoxicity was observed for any of the nanoparticle concentrations tested in the case of G750. Our findings for *S. alba* mustard seeds exposed to GO are only partially comparable to those observed by Ren et al.^62^ on wheat seeds, where lower concentrations of 0.1 g/L caused root growth promotion, but a concentration of 1 g/L caused inhibition, with an explanation of oxidative stress. In another study, the inhibition effect to *Brassica napus* L. seeds, even at 0.1 g/L GO, was reported.^63^ The differences in observations may be attributable not only to variations in plant seeds or exposure conditions but also to the origin of the nanomaterial.

**Table 2:** Germination Index (GI) of *S. Alba* Seeds and Growth Inhibition (*I*) of *D. subspicatus* Exposed to GO and G750 for 72 h^a^,^b^

		*S. alba*	*D. subspicatus*
Sample	*c* (g/L)	GI (%)	*I* (%)
GO	0.01	127.6 ± 17.6	4.9 ± 8.8^b^
	0.1	58.6 ± 7.5	5.2 ± 3.0^b^
	1	46.5 ± 0.7	-
G750	0.01	83.5 ± 7.7	19.3 ± 9.4^a,b^
	0.1	98.6 ± 7.0	41.3 ± 12.4^a^
	1	105.6 ± 18.0	-

a Means that do not share a letter within the same column are statistically significant at *p* < 0.05.

b Different superscripted letters, a and b indicate significant differences (*p* < 0.05) between samples.

Exposure of GO and G750 to freshwater algae *D. subspicatus* provided significantly different results. As demonstrated in Table 2, GO exhibited no inhibition of growth greater than 10% following 72 h of exposure. Conversely, G750 particles demonstrated inhibition at a concentration as low as 0.01 g/L. The explanation for the differences might lie in the particles themselves. The nanoparticles tend to aggregate and then, due to gravitational forces, settle at the bottom of the flask. This process can also result in the formation of aggregates with algae, which may affect their toxic effects since they are present in different water layers.^64^ For instance, this agglomeration phenomenon was observed for G after exposure to freshwater algae *Chlorella pyrenoidosa*, along with a 50% inhibition at a concentration of 0.06 g/L.^65^

### 3.3 Impact of MOC on Prokaryotes and Eukaryotes

As tested, G750 and GO nanoparticles showed beneficial antibiofilm activity against the tested bacteria and low toxicity to eukaryotes; therefore, they were used as nano dopants of MOC, as described in [Sec sec3.1.2]. MOC composites were tested similarly to G750 and GO to provide information about how their properties are affected by the overall matrix of the composite.

We first evaluated the effect of MOC composites on bacterial growth in their vicinity (Figure 8) and on biofilm formation on their surfaces (Figures 9 and 10). All tested MOC composites inhibited both bacterial growth and surface biofilm formation for all three species compared to the reference material (polystyrene). No significant differences were observed between the individual composites, indicating that the addition of G750 or GO did not substantially enhance the antibacterial activity of MOC. This is likely due to the strongly alkaline pH associated with MOC, which is unfavorable for bacterial growth, as most bacteria thrive near neutral pH.^66^ The role of pH in MOC toxicity is crucial regardless of the modification of MOC by C-based materials. Typically, MOC-induced alkalinity reaches pH values of 8–10, which poses a significant challenge for bacterial growth. Extreme alkaline conditions may lead to cell disruptions, conditioning membrane damage, and protein denaturation. Furthermore, elevated pH levels can impair bacterial metabolic activity by altering proton motive force and enzyme functionality, ultimately reducing ATP synthesis and cell viability, and alkaline stress can induce oxidative damage through increased ROS production, further increasing microbial susceptibility.^67^

**Figure 8:**
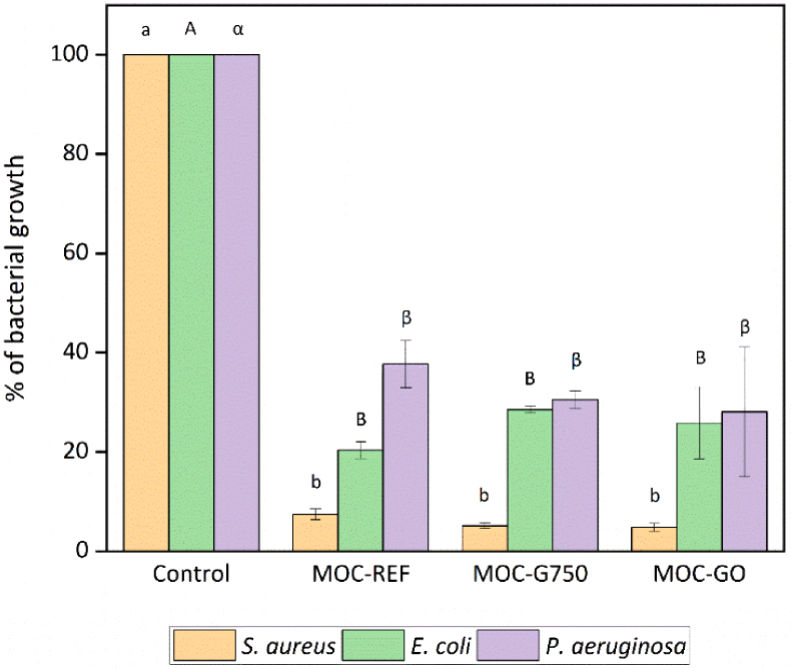
Bacterial growth in the presence of MOC is represented as a percentage of bacterial growth compared to the control. Means that do not share a letter within the same organism are statistically significant at *p* < 0.05.

**Figure 9:**
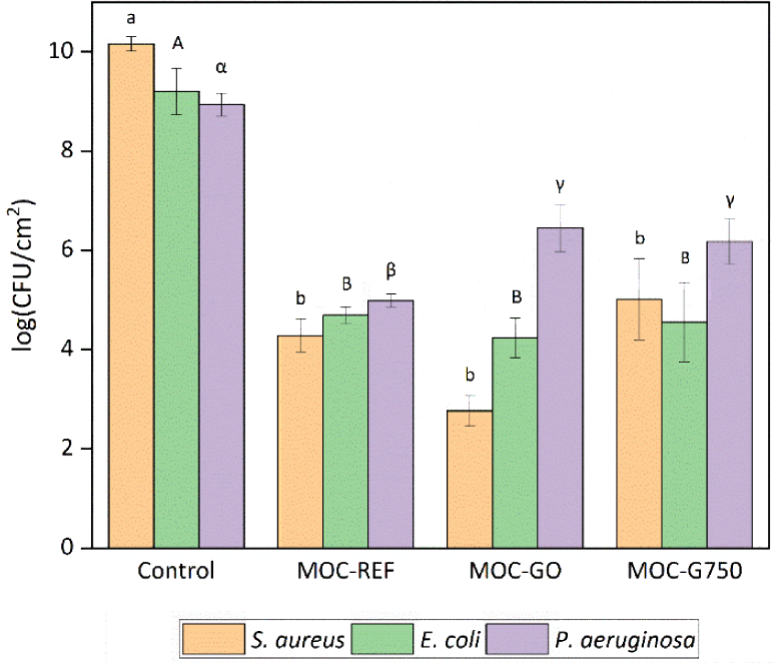
Bacterial biofilm formation on the MOC surface related to 1 cm^2^. Means that do not share a letter within the same organism are statistically significant at *p* < 0.05.

**Figure 10:**
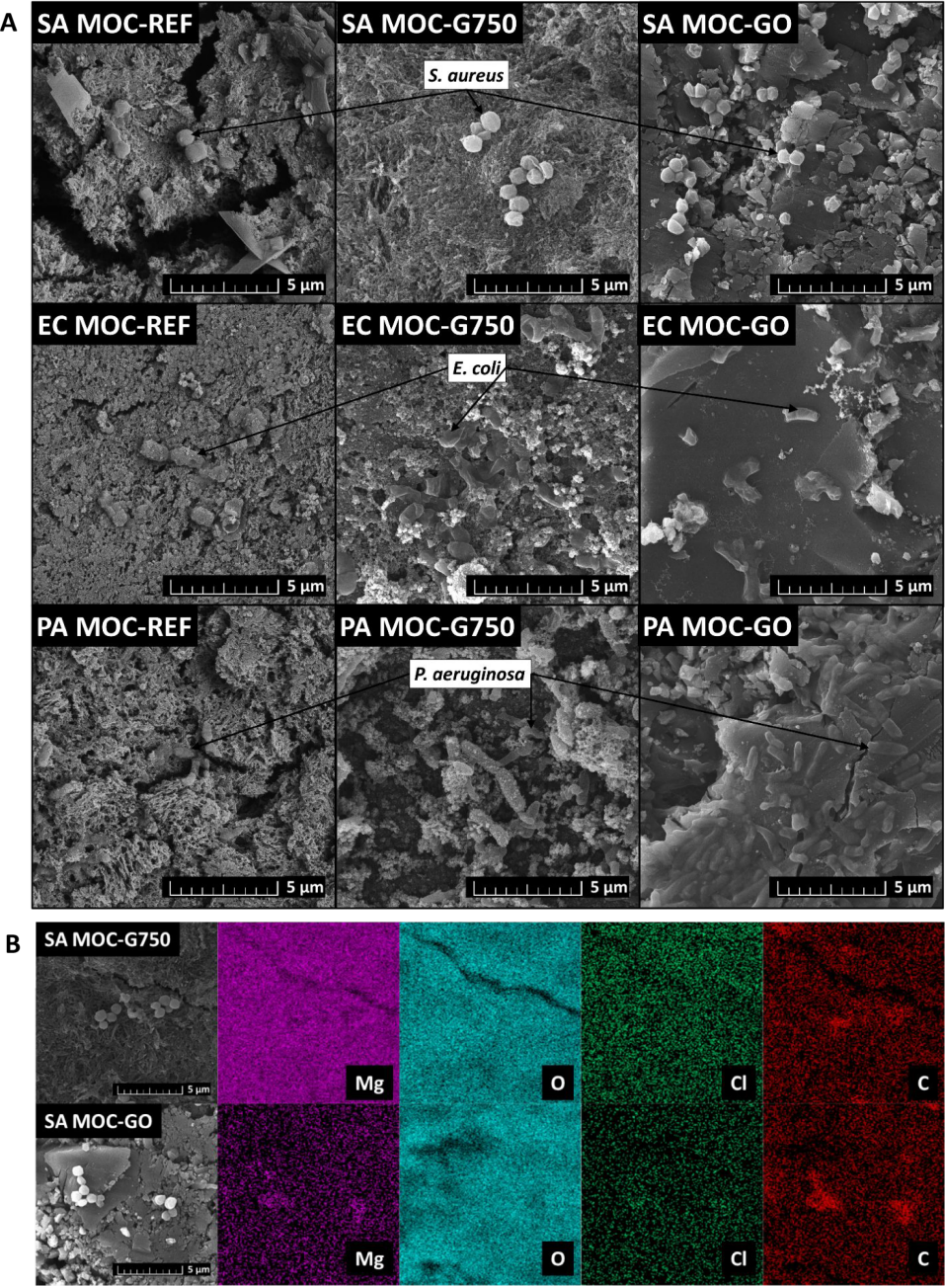
MOC-based composites after exposure to prokaryotes *S. aureus* (SA), *E. coli* (EC), and *P. aeruginosa* (PA): A) SEM micrographs. B) EDS elemental maps of MOC-G750 and MOC-GO composites.

Under such conditions, the potential antimicrobial contribution of G750 and GO addition into the MOC matrix is limited. Further considering the impact of their dispersion in MOC, it is essential to determine their overall availability for interactions with external systems, including biological ones. Inadequate dispersion leads to aggregation, reducing the effective surface area and active sites. Aggregated or unevenly distributed G materials may be overgrown by biofilms, diminishing contact with bacterial cells and thus reducing antimicrobial efficacy.^68^ Similar effects have been observed for metal oxide nanoparticles, where poor dispersion and sample preparation hinder biological activity.^69,70^ Optimizing dispersion protocols is, therefore, critical to maximize the bioavailability and performance of G-based additives in MOC composites. However, since G750 and GO alter the surface roughness of MOC, they may influence microbial adhesion and potentially reduce colonization by other species. Consistent with earlier findings, *P. aeruginosa* showed greater resilience than *S. aureus* and *E. coli*, managing to grow near MOC and adhere to its surface (Figure 10). Still, the impact of MOC was evident—*P. aeruginosa* failed to produce its characteristic pyocyanin pigment, which typically imparts a blue-green hue to the culture. This likely results from the alkaline environment (pH 9–10), which impairs the bacterium’s physiological functions. However, pigment production is a complex, tightly regulated phenomenon dependent on several factors, such as population density and quorum sensing, as pyocyanin serves as an electron shuttle within less aerated biofilm regions. Additionally, environmental factors, mainly temperature and oxygen availability, are crucial;^71^ the tested strain produces peak pyocyanin levels near 32 °C, while our experiments were conducted at 37 °C. Therefore, the absence of pyocyanin production during our test reflects a combination of several conditions, emphasizing the need for a multifactorial perspective when interpreting its production under material exposure and providing the basis for further detailed testing.

Regarding the bacterial ability to adhere to and colonize the MOC surface, the SEM analysis showed a minimal number of cells on the studied surface. In the micrographs shown in Figure 10, it can be seen that all of the prokaryotes underwent visible damage, mainly manifested in a somewhat crumpled surface. The obtained results show suppressed growth of a continuous biofilm, indicating the possible antibacterial effect of the studied samples. In the case of exposure to *S. aureus*, the samples also underwent testing with EDS, showing that in the area of presence of the prokaryote residue, there is an increased amount of carbon detected in both MOC-G750 and MOC-GO. The obtained elemental maps are shown in Figure 10.

The analysis of bacterial interactions with MOC was followed by an analysis of their impact on eukaryotes. The results of the germination index (GI), as determined after exposure to *S. alba* seeds are shown in Figure 11. GI integrates seed germination rate and root elongation measurements, offering a comprehensive evaluation of plant development. While a slight degree of inhibition was observed for GO in combination with MOC at the highest concentration tested (1 g/L), a notable inhibition was observed for MOC-G750 from a concentration as low as 0.1 g/L. Furthermore, a substantial inhibition of growth and germination was observed for the highest concentration of MOC-G750, which can be regarded as highly phytotoxic according to the established GI range. A comparison with the MOC-REF germination index in mustard seeds reveals that the addition of GO does not have a negative effect, as opposed to the result obtained when G750 is added to MOC. MOC-G750 has a direct phytotoxic effect, despite the fact that G750 nanoparticles did not exhibit any phytotoxic effect (Table 2). Therefore, the potentiation of the MOC-G750 composite with respect to *S. alba* seeds can be discussed.

**Figure 11:**
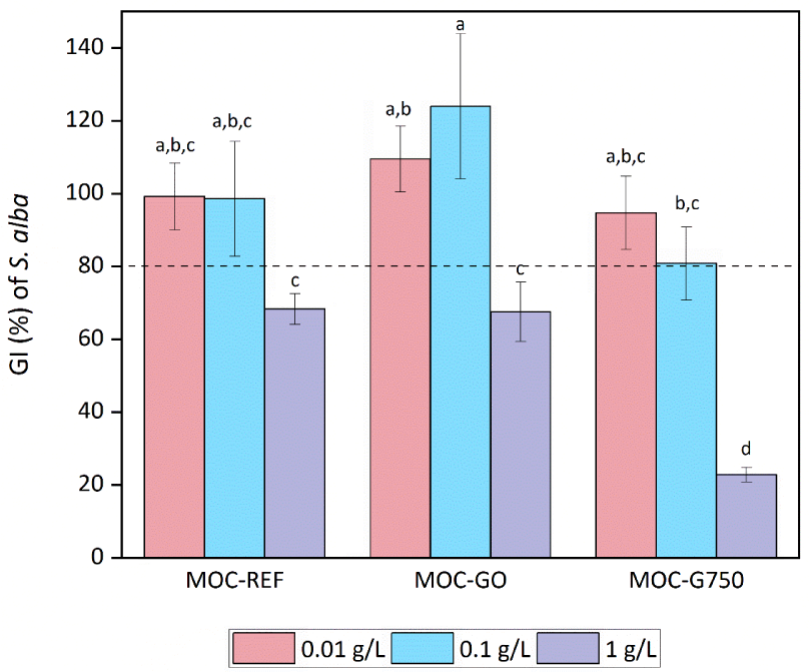
Germination index of *Sinapis alba* seeds after exposure to MOC-REF and MOC composites for 72 h. The dashed line is an indicator of phytotoxicity with a germination index less than 80%.

It is noticeable (Table 4) that addition of GO or G750 nanoparticles to MOC lowered its toxic potential toward saltwater crustaceans *A. salina*. Even at the highest tested concentration of 1 g/L, no significant toxicity was observed for both the composites. Compared with GO or G750 alone (Figure 7), a higher toxic effect would be expected. This is probably because of the low concentration of nanomaterials in the composites or the limited bioavailability of the particles, preventing their interaction with and potential toxicity to *A. salina*. Embedding of carbon nanoparticles within a cementitious matrix causes their physical encapsulation and therefore reduces the potential release into the environment.^72^ Also, the addition of the particles made MOC more stable by reducing its hydrophilic properties, and thus probably prevented its hydration products from leaching out, which may have been the reason for the observed mortality of MOC-REF toward *A. salina*. The use of composites may therefore have the dual advantage of improving the physical–chemical properties of the cement matrix^73^ while also reducing the toxicity to selected organisms. In terms of bioaccumulation, MOC-G750 particles from the highest tested concentration of 1 g/L were found in *A. salina* gut, but without any visible signs of body damage or behavioral changes (Figure S2).

All results for *D. subspicatus* growth inhibition were not statistically significant (Table 3). Only MOC-REF at a concentration of 0.1 g/L caused a higher inhibition than 10%, which could indicate a toxic potential, but the value is still insignificant. A comparison of the inhibition levels observed after exposure to MOC-REF, MOC-GO, and MOC-G750 reveals that the presence of nanomaterial does not influence the toxicity of MOC, despite the significant inhibition caused by G750 itself (Table 2). In order to enhance comprehension of the material effects over time, the exposure period of the algae was extended to 7 days. The growth curves are shown in Figure 12. These curves demonstrate that the algae exhibited no problems with growth in the presence of MOC-REF or MOC-G750, even at the highest concentration tested. Conversely, the exposure revealed a negative effect of MOC-GO on algal growth as early as day four. A potential explanation is the ability of GO to bind iron via its oxygen-containing functional groups, disrupting metabolic processes that are essential for organisms, such as photosynthesis, and the increase of such effects with time.^74^ Also, while composites share the same matrix, the inclusion of G750 or GO particles influences how the material interacts with the environment. For instance, GO’s functional groups may increase its activity over time by creating ROS.^57^

**Table 3:** Results of *Artemia salina* Mortality after 24 h Exposure and Growth Inhibition (*I*) of *Desmodesmus subspicatus* Exposed to MOC-Ref and MOC Composites for 72 h[Table-fn tbl3fn1],[Table-fn tbl3fn2]

		*A. salina*	*D. subspicatus*
Sample	*c* (g/L)	Mortality (%)	*I* (%)
MOC-REF	0.01	13.3 ± 9.4^b^	3.6 ± 6.5
	0.1	23.3 ± 4.7^a,b^	10.8 ± 11.0
	1	43.3 ± 9.4^a^	-
MOC-GO	0.01	3.3 ± 4.7^b^	2.9 ± 0.4
	0.1	3.3 ± 4.7^b^	5.4 ± 3.9
	1	3.3 ± 4.7^b^	-
MOC-G750	0.01	10.0 ± 0.0^b^	2.4 ± 5.6
	0.1	6.7 ± 4.7^b^	0.4 ± 6.8
	1	3.3 ± 4.7^b^	-

i Means that do not share a letter within the same column are statistically significant at *p* < 0.05.

ii Different superscripted letters, a and b, indicate significant differences (*p* < 0.05) between samples.

**Figure 12:**
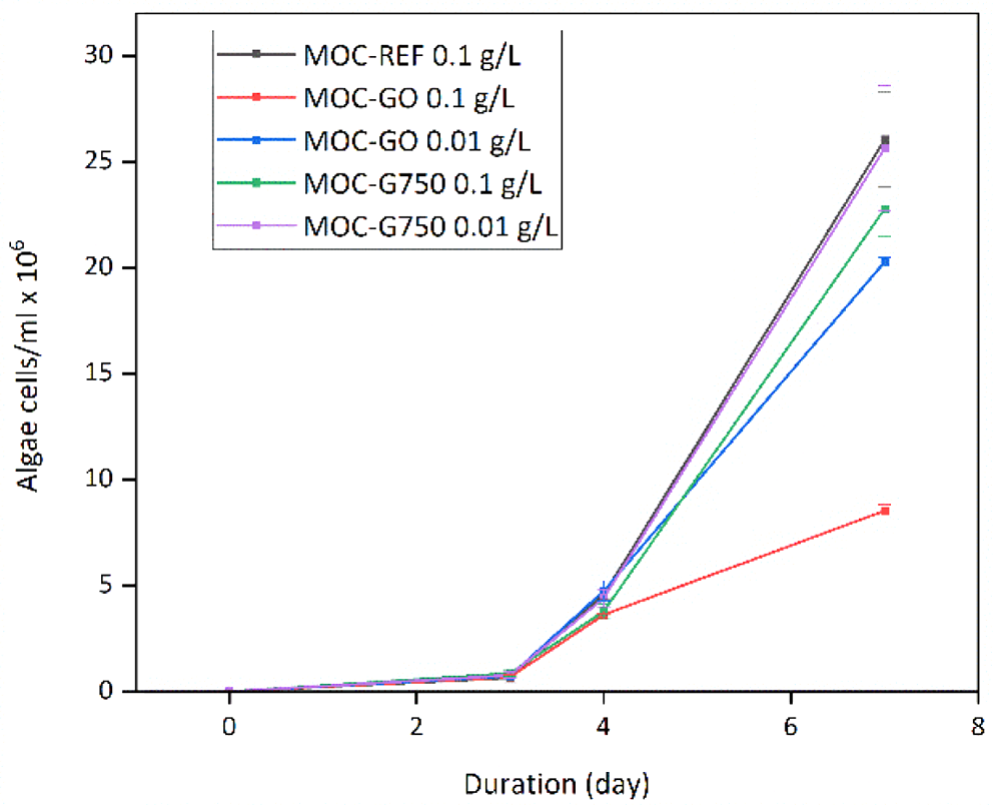
Growth curves of *D. subspicatus* algae in the presence of MOC.

### 3.4 Genotoxic Potential and ROS Production

For a deeper understanding of the impact of the tested materials on organisms, genotoxic potential and influence on ROS production (Table 4) were verified for both G750 and GO nanoparticles and the prepared MOCs, using the highest concentration (1 g/L) tested in this study. Both *in vitro* and *in vivo* genotoxic potential testing revealed no difference in DNA integrity between the control and material-treated samples (Figure 13), indicating that none of the tested materials exhibited genotoxic potential. Our results are in accordance with other recent studies reporting no genotoxic effect of G-based materials, including GO, on microbial DNA.^75,76^ However, it has to be noted that, similar to many other types of nanoparticles, the genotoxic potential of G-based materials and composites may be dose-dependent and should be assessed if higher concentrations are used.

**Table 4:** ROS Generation Increase after the Exposure of Bacteria to G750, GO, and MOCs Expressed in % of Relative Luminescence Units (Compared to Control without Materials)

%RLU						
Strain	Control	G750^a^	GO	MOC-G750	MOC-GO	MOC-REF
SA	100 ± 0.0	39.3 ± 2.6	99.6 ± 9.6	106.4 ± 1.9	111.8 ± 6.3	99.6 ± 5.3
EC	100 ± 0.0	51.5 ± 12.3	88.3 ± 6.6	91.8 ± 7.6	69.5 ± 10.1	56.2 ± 10.4
PA	100 ± 0.0	158 ± 15.2	211.0 ± 17.6	113.7 ± 5.6	132.9 ± 18.4	155.6 ± 13.7
None	100 ± 0.0	37.4 ± 2.1	103.4 ± 16.8	116.8 ± 25.9	107.3 ± 15.1	104.1 ± 15.8

a Due to the luminescence-based nature of the assay, measurements of highly concentrated G solutions—particularly G750—may be systematically underestimated, as their dark color and turbidity absorb emitted light and distort the luminometric signal.

**Figure 13:**
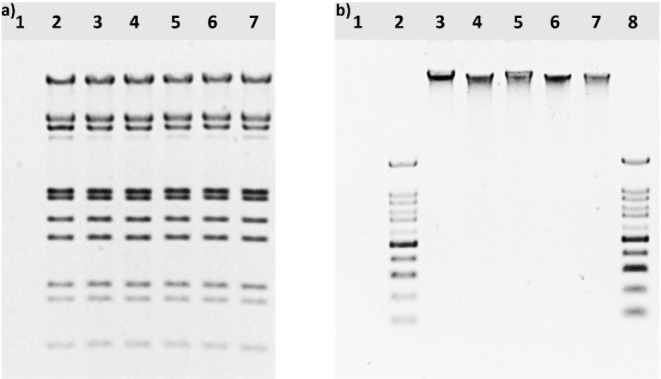
Electrophoretogram of a) *in vitro* genotoxic potential analysis: 1. negative control (NFW), 2. positive control (lambda DNA + NFW), 3. lambda DNA + MOC-REF, 4. lambda DNA + MOC-GO, 5. lambda DNA + MOC-G750, 6. lambda DNA + GO, 7. lambda DNA + G750; b) *in vivo* genotoxic potential analysis: 1. negative control (NFW), 2. and 8. CSL-MDNA-100BP (100 bp DNA ladder; Cleaver Scientific, UK), 3. positive control (DNA + NFW), 4. DNA + MOC-GO, 5. DNA + MOC-G750, 6. DNA + GO, 7. DNA + G750.

Further, we investigated the impact of the materials on reactive oxygen species (ROS) production, as oxidative stress is considered one of the key mechanisms of action of G-based materials.^75^ H_2_O_2_ levels were used as indicators of material-induced oxidative stress in the tested bacterial species, given that H_2_O_2_ possesses the longest half-life among ROS and therefore serves as a reliable marker for cumulative oxidative changes over time.^77^ Due to the luminescence-based nature of the assay, measurements of highly concentrated G750/GO solutions are prone to systematic errors and should be interpreted with caution. This bias arises from the dark coloration and increased turbidity of G-containing solutions, which absorb emitted light and thereby distort the luminometric signal. As a result, relative luminescence unit (RLU) values for GO and especially G750 solutions are significantly underestimated compared to those that would be obtained in a transparent medium. The experiments revealed a notable increase—by several tens of percent—in ROS production in *P. aeruginosa* in the presence of GO and G750. Conversely, ROS levels in *E. coli* were lower than in the control, suggesting that *in vivo* measurement of ROS production reflects not only the oxidative stress directly induced by the material but also the specific stress response of the bacterial cell itself. To further explore this phenomenon, we also assessed ROS production in the absence of bacterial cells. Based on the obtained data, it was determined that ROS formation by the tested G and MOC materials alone is negligible and falls within the range of statistical deviation. These results are consistent with the analysis of genotoxic potential, as ROS production is one of the mechanisms by which G-based materials affect DNA integrity.^75^

## 4 Conclusion

MOC/G-based composites represent a promising and environmentally friendly alternative to conventional cementitious materials. The tested MOC systems, reinforced with G750 and GO, exhibit favorable mechanical and durability properties, including enhanced water resistance-one of the primary limitations of traditional MOC binders. Given their application potential, assessing the ecotoxicological safety of these materials is crucial. We found that while G750 and GO possess antibacterial activity—especially against *S. aureus*—they do not significantly enhance the antibacterial effect of MOC itself, likely due to the inherently high alkalinity of the matrix. Importantly, the tested materials showed no genotoxic potential *in vitro* and *in vivo*, and no significant increase in ROS production was observed, further supporting their biosafety in microbial systems. However, G750, GO, and MOCs exhibited notable species-specific toxicity in eukaryotic organisms. While MOC-GO composites suppressed algal growth after prolonged exposure, MOC-G750 showed stronger phytotoxic effects on *Sinapis alba*. In contrast, both composites displayed reduced toxicity toward *Artemia salina* compared with the nanomaterials alone, likely due to encapsulation within the matrix. These findings indicate that, despite their shared matrix and similar physicochemical properties, MOC-G750 and MOC-GO composites may exert distinct ecological impacts—particularly on eukaryotes. This implies the need for further detailed and robust studies to assess organism-specific responses, nanoparticle bioavailability, and long-term effects. Developing predictive models based on these variables would be key to ensuring the safe application of G-based MOC composites. Following this, upscaling of MOC-G750 and MOC-GO production could offer a competitive and eco-conscious solution for microbial-resistant building components.

## Supplementary Material



## Data Availability

According to open science principles, data can be found at DOI: 10.5281/zenodo.15322187.

## Notes

Fragments of prepared MOC-based composite samples (Figure S1); bioaccumulation of G and MOC particles in *A. salina* gut (Figure S2) (PDF)

## Notes

S.L.: conceptualization, methodology, investigation, data curation, formal analysis, writing – original draft, and writing – review & editing; J.K.: methodology, investigation, visualization, data curation, formal analysis, writing – original draft, and writing – review & editing; V.P.: methodology, investigation, formal analysis, visualization, writing – original draft, and writing – review & editing; A.-M.L.: writing – original draft, investigation, data curation, formal analysis, visualization, and writing – review & editing; A.J.: writing – original draft, investigation, data curation, formal analysis, and writing – review & editing; M.L.: investigation and data curation; R.V.: investigation and data curation; O.J.: project administration, writing – original draft, resources, and supervision

## References

[1] Jankovský O., Lojka M., Lauermannová A.-M., Antončík F., Pavlíková M., Pavlík Z., Sedmidubský D. (2020). Carbon Dioxide Uptake by MOC-Based Materials. Appl. Sci..

[2] Power I. M., Dipple G. M., Francis P. S. (2017). Assessing
the carbon sequestration
potential of magnesium oxychloride cement building materials. Cem. Concr. Compos..

[3] Kastiukas G., Ruan S., Unluer C., Liang S., Zhou X. (2019). Environmental
Assessment of Magnesium Oxychloride Cement Samples: A Case Study in
Europe. Sustainability.

[4] Singh A., Kumar R., Goel P. (2021). Factors influencing strength of magnesium
oxychloride cement. Constr. Build. Mater..

[5] Dehua D., Chuanmei Z. (1999). The formation mechanism
of the hydrate phases in magnesium
oxychloride cement. Cem. Concr. Res..

[6] Wang Y., Wang H., Zhang L., Peng L., Lyu S., Huang L. (2024). Optimization
of raw-material ratios and curing temperature of magnesium
oxychloride cement. Constr. Build. Mater..

[7] Sugimoto K., Dinnebier R. E., Hanson J. C. (2007). Structures of three dehydration products
of bischofite from in situ synchrotron powder diffraction data (MgCl2.nH2O;
n = 1, 2, 4). Acta Crystallogr., Sect. B.

[8] Zhang M., Yu H., Ma H., Wu C., Zhu B., Li Y., Li L., Kang Y., Ding Z. (2025). Effect of 5·1·8 whiskers
on the mechanical properties and microstructure of magnesium oxychloride
cement. Composites, Part B.

[9] Li K., Wang Y., Zhang X., Wang X., Zhang A. (2021). Raw material
ratio optimization of magnesium oxychloride cement using response
surface method. Constr. Build. Mater..

[10] Li Z., Chau C. K. (2007). Influence of Molar
Ratios on Properties of Magnesium
Oxychloride Cement. Cem. Concr. Res..

[11] Singh, N. B. ; Shukla, S. K. Chapter 3 - Properties of two-dimensional nanomaterials. In Two-Dimensional Nanostructures for Biomedical Technology, Khan, R. ; Barua, S. , Eds.; Elsevier, 2020, pp. 73–100.

[12] Shanmugam V., Mensah R. A., Babu K., Gawusu S., Chanda A., Tu Y., Neisiany R. E., Försth M., Sas G., Das O. (2022). A Review of
the Synthesis, Properties, and Applications of 2D Materials. Part. Syst. Charact..

[13] Liu B., Zhou K. (2019). Recent progress
on graphene-analogous 2D nanomaterials: Properties,
modeling and applications. Prog. Mater. Sci..

[14] Fatma, I. ; Assad, H. ; Kumar, A. Introduction to Two-Dimensional Nanomaterials. In Two-Dimensional Nanomaterials-Based Polymer Nanocomposites, Pandey, M. ; Desmukh, K. ; Hussain, C. M. , Eds.; Scrivener Publishing, 2024, pp. 1–45.

[15] Fan Y., Zhang G., Li Y. (2022). Study on graphene
oxide reinforced
magnesium phosphate cement composites. Constr.
Build. Mater..

[16] Lu Z., Hou D., Ma H., Fan T., Li Z. (2016). Effects of graphene
oxide on the properties and microstructures of the magnesium potassium
phosphate cement paste. Constr. Build. Mater..

[17] Jiang L., Liu Z., Yu Y., Ben X. (2021). The effect of graphene on the conductivity
of magnesium sulfate cement. Constr. Build.
Mater..

[18] Du Y., Yang J., Skariah
Thomas B., Li L., Li H., Mohamed
Shaban W., Tung Chong W. (2020). Influence of hybrid graphene oxide/carbon
nanotubes on the mechanical properties and microstructure of magnesium
potassium phosphate cement paste. Constr. Build.
Mater..

[19] Jiříčková A., Lauermannová A.-M., Jankovský O., Lojka M., Záleská M., Pivák A., Pavlíková M., Merglová A., Pavlik Z. (2024). Impact of nano-dopants on the mechanical and physical
properties of magnesium oxychloride cement composites – Experimental
assessment. J. Build. Eng..

[20] Freixa A., Acuña V., Sanchís J., Farré M., Barceló D., Sabater S. (2018). Ecotoxicological effects
of carbon
based nanomaterials in aquatic organisms. Sci.
Total Environ..

[21] Gamoń F., Ziembińska-Buczyńska A., Łukowiec D., Tomaszewski M. (2023). Ecotoxicity of selected carbon-based
nanomaterials. Int. J. Environ. Sci. Technol..

[22] Seabra A. B., Paula A. J., de Lima R., Alves O. L., Durán N. (2014). Nanotoxicity
of Graphene and Graphene Oxide. Chem. Res. Toxicol..

[23] Akhavan O., Ghaderi E. (2010). Toxicity of Graphene
and Graphene Oxide Nanowalls Against
Bacteria. ACS Nano.

[24] Hu W., Peng C., Luo W., Lv M., Li X., Li D., Huang Q., Fan C. (2010). Graphene-Based
Antibacterial Paper. ACS Nano.

[25] Bykkam S., Rao K., Chakra S., Thunugunta T. (2013). Synthesis and characterization of
graphene oxide and its antibacterial activity against *Klebseilla* and *Staphylococus*. Int. J.
Adv. Biotechnol. Res..

[26] Feng L., Liu Z. (2011). Graphene in Biomedicine: Opportunities and Challenges. Nanomedicine.

[27] Breijyeh Z., Jubeh B., Karaman R. (2020). Resistance of Gram-Negative Bacteria
to Current Antibacterial Agents and Approaches to Resolve It. Molecules.

[28] Liu J., Tang J., Gooding J. J. (2012). Strategies
for chemical modification
of graphene and applications of chemically modified graphene. J. Mater. Chem..

[29] Malina T., Maršálková E., Holá K., Tuček J., Scheibe M., Zbořil R., Maršálek B. (2019). Toxicity of graphene oxide against
algae and cyanobacteria: Nanoblade-morphology-induced mechanical injury
and self-protection mechanism. Carbon.

[30] Ouyang S., Hu X., Zhou Q. (2015). Envelopment–Internalization
Synergistic Effects
and Metabolic Mechanisms of Graphene Oxide on Single-Cell Chlorella
vulgaris Are Dependent on the Nanomaterial Particle Size. ACS Appl. Mater. Interfaces.

[31] Pikula K., Johari S. A., Santos-Oliveira R., Golokhvast K. (2023). The Comparative
Toxic Impact Assessment of Carbon Nanotubes, Fullerene, Graphene,
and Graphene Oxide on Marine Microalgae Porphyridium purpureum. Toxics.

[32] Hazeem L. J., Bououdina M., Dewailly E., Slomianny C., Barras A., Coffinier Y., Szunerits S., Boukherroub R. (2017). Toxicity effect of graphene oxide
on growth and photosynthetic
pigment of the marine alga Picochlorum sp. during different growth
stages. Environ. Sci. Pollut. Res. Int..

[33] Nogueira P. F. M., Nakabayashi D., Zucolotto V. (2015). The effects of graphene oxide on
green algae Raphidocelis subcapitata. Aquat.
Toxicol..

[34] Matos D., Almeida S. F. P., Marques P. A. A. P., Pinto S., Figueira E. (2023). Effects of
Graphene Oxide Nanosheets in Freshwater Biofilms. Molecules.

[35] Wang Q., Li C., Wang Y., Que X. (2019). Phytotoxicity of Graphene Family
Nanomaterials and Its Mechanisms: A Review. Front. Chem..

[36] Liu S., Wei H., Li Z., Li S., Yan H., He Y., Tian Z. (2015). Effects of Graphene on Germination and Seedling Morphology
in Rice. J. Nanosci. Nanotechnol..

[37] Chen J., Yang L., Li S., Ding W. (2018). Various Physiological
Response to Graphene Oxide and Amine-Functionalized Graphene Oxide
in Wheat (*Triticum aestivum*). Molecules.

[38] Yin L., Wang Z., Wang S., Xu W., Bao H. (2018). Effects of
Graphene Oxide and/or Cd2+ on Seed Germination, Seedling Growth, and
Uptake to Cd2+ in Solution Culture. Water, Air,
Soil Pollut..

[39] Begum P., Ikhtiari R., Fugetsu B. (2011). Graphene phytotoxicity
in the seedling
stage of cabbage, tomato, red spinach, and lettuce. Carbon.

[40] He Y., Hu R., Zhong Y., Zhao X., Chen Q., Zhu H. (2018). Graphene oxide
as a water transporter promoting germination of plants in soil. Nano Res..

[41] Zhang M., Gao B., Chen J., Li Y. (2015). Effects of graphene on seed germination
and seedling growth. J. Nanopart. Res..

[42] Németh I., László K., Bulátkó A., Vaszita E., Molnár M. (2023). Ecotoxicity
Assessment of Graphene Oxides Using Test
Organisms from Three Hierarchical Trophic Levels to Evaluate Their
Potential Environmental Risk. Nanomaterials.

[43] Fekete-Kertész I., László K., Terebesi C., Gyarmati B. S., Farah S., Márton R., Molnár M. (2020). Ecotoxicity Assessment of Graphene
Oxide by Daphnia magna through a Multimarker Approach from the Molecular
to the Physiological Level including Behavioral Changes. Nanomaterials.

[44] Souza J. P., Venturini F. P., Santos F., Zucolotto V. (2018). Chronic toxicity
in Ceriodaphnia dubia induced by graphene oxide. Chemosphere.

[45] Malhotra N., Villaflores O. B., Audira G., Siregar P., Lee J.-S., Ger T.-R., Hsiao C.-D. (2020). Toxicity Studies on Graphene-Based
Nanomaterials in Aquatic Organisms: Current Understanding. Molecules.

[46] Jankovský O., Lojka M., Lauermannová A.-M., Antončík F., Pavlíková M., Záleská M., Pavlik Z., Pivák A., Sedmidubský D. (2020). Towards novel
building materials: High-strength nanocomposites based on graphene,
graphite oxide and magnesium oxychloride. Appl.
Mater. Today.

[47] Sobarzo-Bernal O., Gómez-Merino F. C., Alcántar-González G., Saucedo-Veloz C., Trejo-Téllez L.
I. (2021). Biostimulant Effects
of Cerium on Seed Germination and Initial Growth of Tomato Seedlings. Agronomy.

[48] Jiříčková A., Lauermannová A.-M., Jankovský O., Fathi J., Záleská M., Pivák A., Pavlíková M., Jeremiáš M., Pavlik Z. (2023). Utilization of waste carbon spheres in Magnesium Oxychloride
Cement. Case Stud. Constr. Mater..

[49] Lauermannová A.-M., Pavlíková M., Pavlík Z., Pivák A., Jiříčková A., Sklenka J., Záleská M., Růžička K., Jankovský O. (2022). Magnesium oxychloride cement with phase change material:
Novel environmentally-friendly composites for heat storage. J. Mater. Res. Technol..

[50] Lencova S., Stindlova M., Havlickova K., Jencova V., Peroutka V., Navratilova K., Zdenkova K., Stiborova H., Hauzerova S., Kostakova E. K., Jankovsky O., Kejzlar P., Lukas D., Demnerova K. (2024). Influence
of Fiber Diameter of Polycaprolactone Nanofibrous Materials on Biofilm
Formation and Retention of Bacterial Cells. ACS Appl. Mater. Interfaces.

[51] Lencova S., Kofronova J., Peroutka V., Lauermannova A. M., Jirickova A., Lojka M., Jankovsky O., Vurm R. (2025). Ecotoxicological assessment
of MWCNT-reinforced MOC composites: Impacts
on model bacteria and eukaryotes with environmental relevance. Environ. Sci.: Nano.

[52] Gies V., Zou S. (2018). Systematic toxicity investigation of graphene oxide: Evaluation of
assay selection, cell type, exposure period and flake size. Tox. Res..

[53] da
Cruz Nizer W. S., Allison Kira N., Adams Madison E., Vargas Mario A., Ahmed D., Beaulieu C., Raju D., Cassol E., Howell P. L., Overhage J. (2024). The role of exopolysaccharides
Psl and Pel in resistance of *Pseudomonas aeruginosa* to the oxidative stressors sodium hypochlorite and hydrogen peroxide. Microbiol. Spectrum.

[54] Melaugh G., Martinez V. A., Baker P., Hill P. J., Howell P. L., Wozniak D. J., Allen R. J. (2023). Distinct types of
multicellular aggregates
in *Pseudomonas aeruginosa* liquid cultures. Npj Biofilms Microbiomes.

[55] Gaylarde C., Silva M., Warscheid T. (2003). Microbial impact on building materials:
An overview. Mater. Struct..

[56] Krishnamoorthy K., Umasuthan N., Mohan R., Lee J., Kim S.-J. (2012). Antibacterial
Activity of Graphene Oxide Nanosheets. Sci.
Adv. Mater..

[57] Yadav N., Dubey A., Shukla S., Saini C. P., Gupta G., Priyadarshini R., Lochab B. (2017). Graphene Oxide-Coated Surface: Inhibition
of Bacterial Biofilm Formation due to Specific Surface–Interface
Interactions. ACS Omega.

[58] Mesarič T., Gambardella C., Milivojević T., Faimali M., Drobne D., Falugi C., Makovec D., Jemec Kokalj A., Sepcic K. (2015). High surface adsorption
properties of carbon-based
nanomaterials are responsible for mortality, swimming inhibition,
and biochemical responses in *Artemia salina* larvae. Aquat. Toxicol..

[59] Zhu S., Luo F., Chen W., Zhu B., Wang G. (2017). Toxicity evaluation
of graphene oxide on cysts and three larval stages of Artemia salina. Sci. Total Environ..

[60] Lu J., Zhu X., Tian S., Lv X., Chen Z., Jiang Y., Liao X., Cai Z., Chen B. (2018). Graphene oxide in the
marine environment: Toxicity to *Artemia salina* with
and without the presence of Phe and Cd2+. Chemosphere.

[61] Pretti C., Oliva M., Pietro R. D., Monni G., Cevasco G., Chiellini F., Pomelli C., Chiappe C. (2014). Ecotoxicity of pristine
graphene to marine organisms. Ecotoxicol. Environ.
Saf..

[62] Ren W., Chang H., Li L., Teng Y. (2020). Effect of Graphene
Oxide on Growth of Wheat Seedlings: Insights from Oxidative Stress
and Physiological Flux. Bull. Environ. Contam.
Toxicol..

[63] Cheng F., Liu Y.-F., Lu G.-Y., Zhang X.-K., Xie L.-L., Yuan C.-F., Xu B.-B. (2016). Graphene oxide modulates
root growth
of Brassica napus L. and regulates ABA and IAA concentration. J. Plant Physiol..

[64] Lv B., Wang C., Hou J., Wang P., Miao L., Li Y., Ao Y., Yang Y., You G., Xu Y. (2016). Influence
of shear forces on the aggregation and sedimentation behavior of cerium
dioxide (CeO2) nanoparticles under different hydrochemical conditions. J. Nanopart. Res..

[65] Zhao J., Cao X., Wang Z., Dai Y., Xing B. (2017). Mechanistic understanding
toward the toxicity of graphene-family materials to freshwater algae. Water Res..

[66] Jin Q., Kirk M. F. (2018). pH as a Primary Control in Environmental Microbiology:
1. Thermodynamic Perspective. Front. Environ.
Sci..

[67] Maier A., Manea D. L. (2022). Perspective of Using Magnesium Oxychloride
Cement (MOC)
and Wood as a Composite Building Material: A Bibliometric Literature
Review. Materials.

[68] Anand A., Unnikrishnan B., Wei S. C., Chou C. P., Zhang L. Z., Huang C. C. (2019). Graphene
oxide and carbon dots as broad-spectrum antimicrobial
agents – a minireview. Nanoscale Horiz..

[69] Augustyniak A., Jablonska J., Cendrowski K., Głowacka A., Stephan D., Mijowska E., Sikora P. (2022). Investigating the release
of ZnO nanoparticles from cement mortars on microbiological models. Appl. Nanosci..

[70] Sikora P., Augustyniak A., Cendrowski K., Nawrotek P., Mijowska E. (2018). Antimicrobial
Activity of Al2O3, CuO, Fe3O4, and ZnO Nanoparticles in Scope of Their
Further Application in Cement-Based Building Materials. Nanomaterials.

[71] Cezairliyan B., Vinayavekhin N., Grenfell-Lee D., Yuen G. J., Saghatelian A., Ausubel F. M. (2013). Identification of *Pseudomonas aeruginosa* Phenazines that Kill Caenorhabditis elegans. PLoS Pathog..

[72] Goldmann E., Kudlek E., Bialas O., Górski M., Adamiak M., Klemczak B. (2025). Environmental Toxicity of Cement
Nanocomposites Reinforced with Carbon Nanotubes. Materials.

[73] Gao Y., Pham V. H., Weidman J., Kim K.-J., Spaulding R. E., Wang C., Matranga C. S. (2024). High-performance
cementitious composites
containing nanostructured carbon additives made from charred coal
fines. Sci. Rep..

[74] Yu Q., Zhang B., Li J., Du T., Yi X., Li M., Chen W., Alvarez P. J. J. (2017). Graphene oxide significantly inhibits
cell growth at sublethal concentrations by causing extracellular iron
deficiency. Nanotoxicology.

[75] Domenech J., Rodríguez-Garraus A., López de Cerain A., Azqueta A., Catalán J. (2022). Genotoxicity
of Graphene-Based Materials. Nanomaterials.

[76] Aunkor M. T. H., Raihan T., Prodhan S. H., Metselaar H. S. C., Malik S. U. F., Azad A. K. (2020). Antibacterial activity
of graphene
oxide nanosheet against multidrug resistant superbugs isolated from
infected patients. R. Soc. Open Sci..

[77] Seixas A. F., Quendera A. P., Sousa J. P., Silva A. F. Q., Arraiano C. M., Andrade J. M. (2022). Bacterial Response
to Oxidative Stress and RNA Oxidation. Front.
Genet..

