# Supplementary material for: Low Ecotoxicological Impact of Magnesium Oxychloride Cement Composites Doped with 2D Carbon-Based Nanoadditives
Source: ACS Omega. 2025 Jul 31;10(31):35013–28. doi: 10.1021/acsomega.5c04437 (PMC12355315; doi:10.1021/acsomega.5c04437)

SUPPORTING INFORMATION

Low Ecotoxicological Impact of Magnesium Oxychloride Cement Composites Doped with 2D Carbon-Based Nanoadditives

Simona Lencova[*1,2], Jana Kofronova[2,3], Vaclav Peroutka[1], Anna-Marie Lauermannova[2], Adela Jirickova[2], Michal Lojka[2], Radek Vurm[2], Ondrej Jankovsky[2]

[1] Department of Biochemistry and Microbiology, University of Chemistry and Technology, Prague, Czech Republic

[2] Department of Inorganic Chemistry, University of Chemistry and Technology Prague, Czech Republic

[3] Department of Environmental Chemistry, University of Chemistry and Technology, Prague, Czech Republic

[*]Corresponding author: Simona Lencova, lencovas@vscht.cz

Figure S1: Fragments of prepared MOC-based composite samples

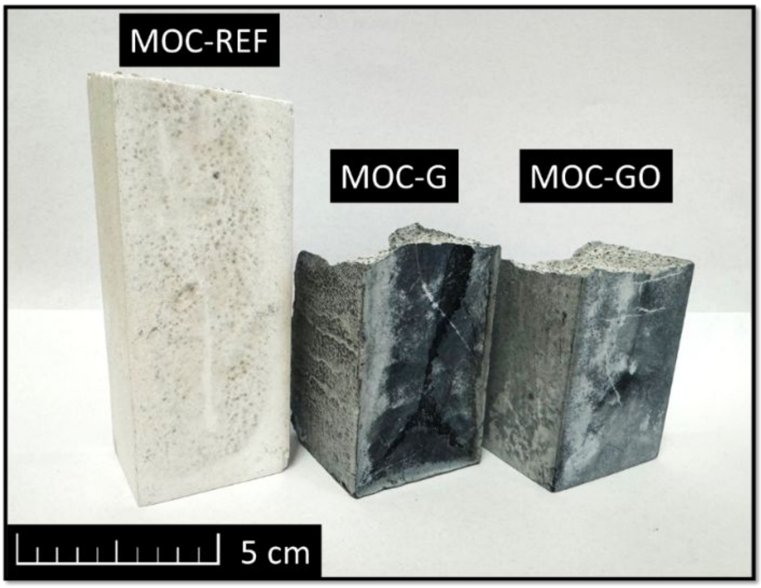

Figure S2: G750 (a), GO (b), MOC-REF (c), MOC-G750 (d), and MOC-GO (e) samples (1 g/L) bioaccumulation in *A. salina*; no visible signs of body damage or behaviour changes were observed.

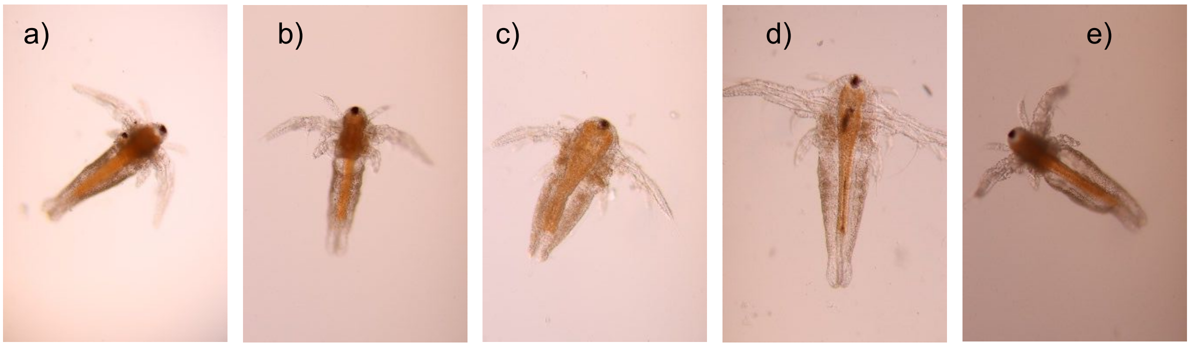

Supplement: Supplementary file 1 [file ao5c04437_si_001.pdf]
